# Supplementary figures and images for: Trends in adverse perinatal outcomes and associated hospitalisations, emergency department presentations, and healthcare costs from birth to early childhood in the Northern Territory, Australia: A two-decade population-based study
Source: PLOS Glob Public Health. 2025 Aug 7;5(8):e0004985. doi: 10.1371/journal.pgph.0004985 (PMC12331054; doi:10.1371/journal.pgph.0004985)

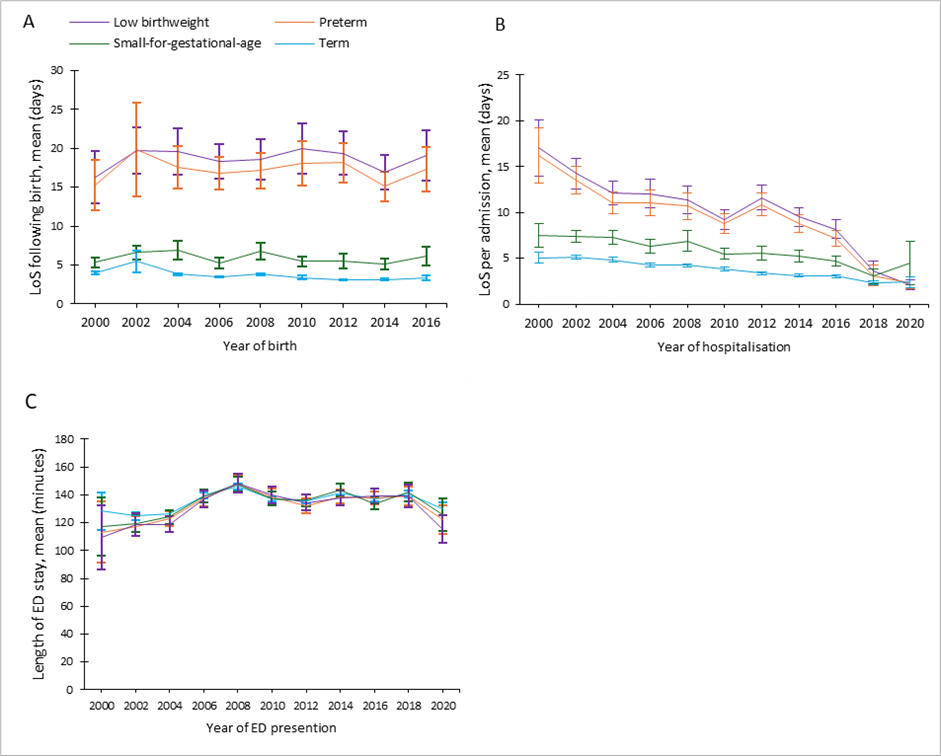

Supplement: S1 Fig — A. Birth hospitalisation, B. Subsequent hospitalisation, and C. ED presentation. (TIF) [file pgph.0004985.s002.tif]

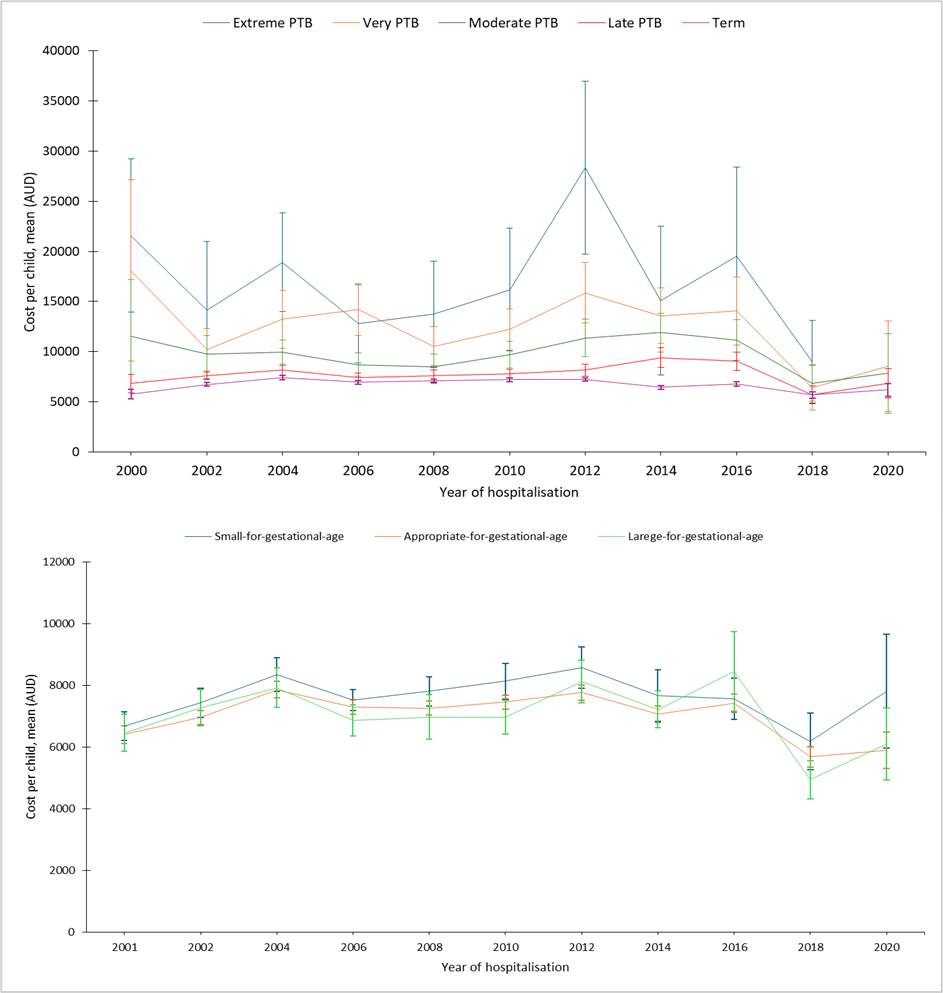

Supplement: S2 Fig — PTB: Preterm birth (TIF) [file pgph.0004985.s003.tif]

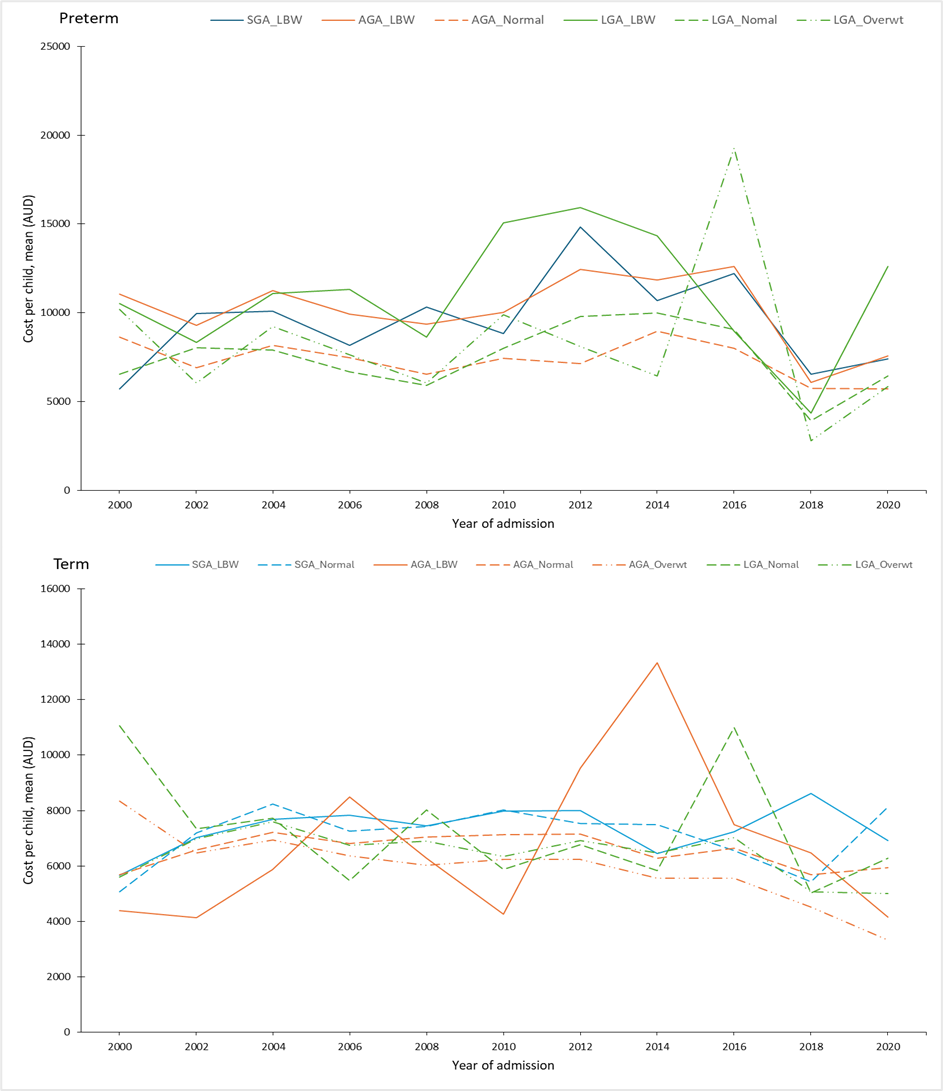

Supplement: S3 Fig — SAG-LBW: Small-for-gestational-age and low birthweight. AGA-LBW: Appropriate-for-gestational-age and low birthweight. AGA-Normal: Appropriate-for-gestational-age and normal birthweight. LGA-LBW: Large-for-gestational-age and low birthweight. LGA-Normal: Large-for-gestational-age and normal birthweight. LGA-Overwt: Large-for-gestational-age and overweight. (TIF) [file pgph.0004985.s004.tif]

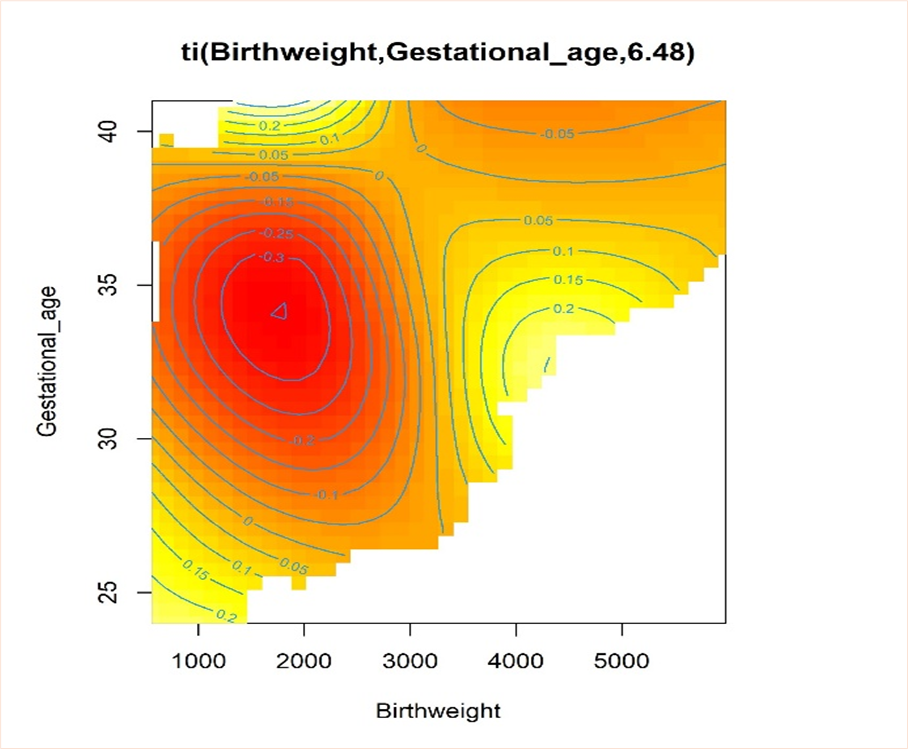

Supplement: S4 Fig — (TIF) [file pgph.0004985.s005.tif]

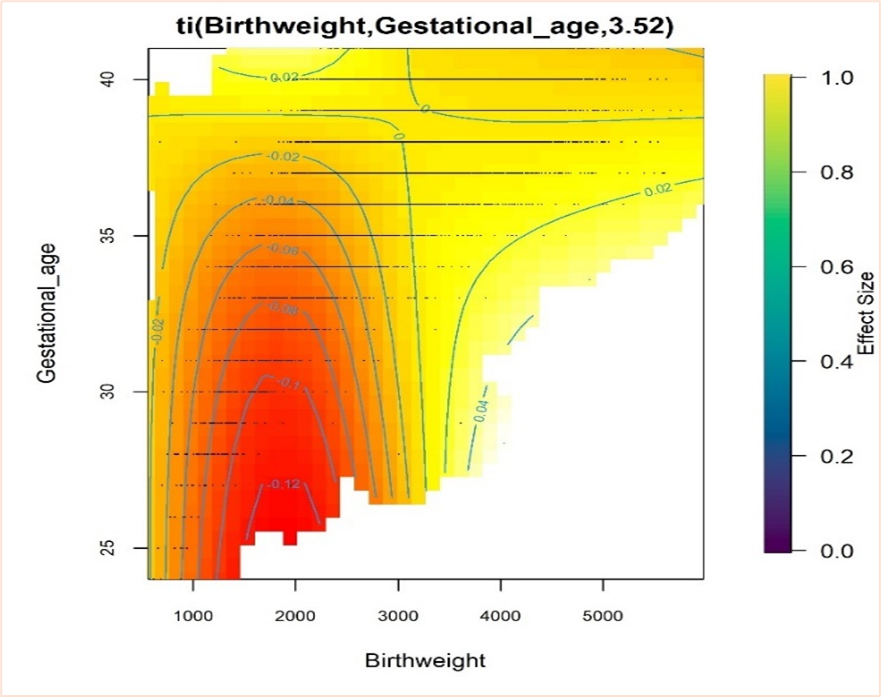

Supplement: S5 Fig — (TIF) [file pgph.0004985.s006.tif]
