# Supplementary material for: Trends in adverse perinatal outcomes and associated hospitalisations, emergency department presentations, and healthcare costs from birth to early childhood in the Northern Territory, Australia: A two-decade population-based study
Source: PLOS Glob Public Health. 2025 Aug 7;5(8):e0004985. doi: 10.1371/journal.pgph.0004985 (PMC12331054; doi:10.1371/journal.pgph.0004985)
Supplement: S1 Table — (DOCX) [file pgph.0004985.s007.docx]

**S1 Table. Cost of hospitalisation by adverse perinatal outcomes and years from birth to age five, NT, Australia, 2000**–**2020.**

| **Year of hospitalisation** | | **Cost per child, median (IQR) (AUD)** | | | | | | | |
| --- | --- | --- | --- | --- | --- | --- | --- | --- | --- |
|  |  | **Overall** | **Term** | **Term with no LBW and SGA** | **Preterm + LBW + SGA** | **At least one adverse perinatal outcome** | **PTB** | **SGA** | **LBW** |
| 2000 | | 4,377 (3,527-6,992) | 3,527 (3,527-5,263) | 3,527 (3,527- 5,640) | 3,808 (3,667-6,623) | 5,234 (3,527-7.753) | 6,581 (3,808-10,162) | 3807 (3,527-5,640) | 5,234 (3,807-9,955) |
| 2001 | | 5,168 (3,484-7,658) | 4,825 (3,484-7,238) | 4,323 (3,484-7,238) | 5,537 (3,761-9,832) | 5,170 (3,760-7,658) | 5,780 (3,761-9,832) | 5,170 (3,761-7,658) | 5,428 (3,761-9,832) |
| 2002 | | 5,575 (3,646-7,984) | 5,411 (3,646-7,575) | 5,411 (3,646-7,575) | 7,574 (6,022-10,467) | 6,403 (3,930-8,468) | 6,803(3,936-10,289) | 6,269 (3,880-8,014) | 6,810 (3,936-10,289) |
| 2003 | | 5,764 (3,851-8,452) | 5,714 (3,851-8,000) | 5,714 (3,851-8,000) | 8,000 (5,955-12,936) | 6,539 (4,039-8,471) | 7,184(4,156-10,345) | 6,539 (3,851-8,472) | 7,247 (4,314-10,707) |
| 2004 | | 6,462 (3,968-8,721) | 5,937 (3,967-8,656) | 5887 (3,967-8,242) | 7,465 (5,044-10,630) | 7,402 (4,282-9,487) | 7,989 (4,282-11,196) | 7,221 (4,080-9,215) | 7,810 (4,308-11,196) |
| 2005 | | 5,998 (4,042-8,886) | 5,998 (3,851-8,398) | 5,747 (3,700-8,398) | 8,140 (5,146-11,408) | 6,480 (4,042-9,389) | 7,542 (4,363-11,408). | 6,091 (4,042-9,139) | 7,349 (4,364-10,889) |
| 2006 | | 5,489 (4,021-8,437) | 5,039 (3,728-8,437) | 5,039 (3,718-8,437) | 7,898 (4,384-10,097) | 6,027 (4,061-8,882) | 7,577 (4,384-10,115) | 6,027 (4,061-8,927) | 7,577 (4,384-11,040) |
| 2007 | | 5,734 (3,986-8,574) | 5,424 (3,709-8,419) | 5,413 (3,709-8,419) | 7,560 (4,701-11,882) | 6,013 (4,052-8,907) | 7,367 (4,375-10,615) | 6,013 (4,052-8,908) | 7,541 (4,374-10,733) |
| 2008 | | 5,245 (3,716-8,433) | 5,041 (3,716-8,433) | 5,036 (3,716-8,433) | 8,174 (4,371-15,086) | 5,866 (4,019-8,433) | 6,080 (4,382-9,669) | 6,023 (3,878-8,922) | 6,516 (4,382-11,029) |
| 2009 | | 5,734 (4,096-8,630) | 5,451 (3,968-8,630) | 5,345 (3,909-8,629) | 7,272 (4,484-11,723) | 6,164 (4,154-9,130) | 7,142 (4,484-11,289) | 6,164 (4,154-9,130) | 6,783 (4,484-11,236) |
| 2010 | | 5,679 (4,219-8,887) | 5,389 (3,927-8,888) | 5,406 (3,927-8,887) | 7,981 (4,618-10,503) | 6,030 (4,278-9,081) | 7,024 (4,618-10,614) | 6,289 (4,278-9,029) | 7,258 (4,574-10,591) |
| 2011 | | 5,683 (4,242-8,938) | 5,343 (3,938-8,937) | 5,338 (3,938-8,718) | 8,087 (4,644-12,141) | 6,384 (4,302-9,763) | 7,531 (4,644-12,141) | 6,384 (4,302-9,746) | 7,384 (4,644-12,237) |
| 2012 | | 5,954 (4,273-9,002) | 5,633 (3,977-9,002) | 5,376 (3,967-9,002) | 9,002 (5,554-14,557) | 6,430 (4,333-10,039) | 8,084 (4,678-12,228) | 6,430 (4,333-9,524) | 8,087 (4,677-12,326) |
| 2013 | | 5,541 (3,768-8,818) | 5,066 (3,557-8,567) | 5,066 (3,557- 8,688) | 7,214 (5,864-13,825) | 6,391 (3,869-9,768) | 7,224 (4,978-13,825) | 6,309 (3,835-8,818) | 7,214 (4,733-13,825) |
| 2014 | | 5,358 (3,832-8,081) | 4,839 (3,698-7,603) | 4,839 (3,695-7,481) | 8,081 (5,180-12,386) | 6,442 (4,104-9,620) | 8,345 (4,839-13,564) | 5,608 (4,105-8,808) | 7,634 (4,839-11,925) |
| 2015 | | 5,186 (4,234-8,165) | 4,994 (4,117-7,685) | 4,994 (4,054-7,685) | 9,887 (6,219-10,278) | 6,552 (4,310-9,887) | 8,278 (4,834-13,641) | 5,692 (4,310-8,837) | 8,061 (4,994-13,641) |
| 2016 | | 5,349 (3,526-8,239) | 4,939 (3,456-7,834) | 4,939 (3,321-7,842) | 7,401 (4,530-12,015) | 5,978 (4,299-9,615) | 7,601 (4,530-12,016) | 5,711 (4,309-8,246) | 7,343 (4,752 -10,846) |
| 2017 | | 4,729 (2,590-6,397) | 4,729 (2,501-6,197) | 4,729 (2,435-6,323) | 5,496 (3,425-6,532) | 4,849 (2,687-6,532) | 5,123 (3,064-6,954) | 4,849 (2,695-6,954) | 4,960 (3,425-6,765) |
| 2018 | | 4,639 (2,062-6,144) | 4,639 (2,053-6,143) | 4,639 (2,053-6,143) | 5,963 (3,654-9,569) | 4,660 (2,088-6,143) | 4,682 (2,490-7,207) | 4,773 (2,039-6,421) | 5,291 (2,289-8,936) |
| 2019 | | 5,208 (2,467-6,638) | 5.177 (2,464-6,578) | 4,634 (2,466-6,407) | 5,808 (5,369-7,400) | 5,602 (2,467-6,806) | 5,557 (2,994-6,806) | 5,807 (3,011-6,964) | 5,681 (3,212-6,964) |
| 2020 | | 5,703 (2,533-6,808) | 5,468 (2,533-6,581) | 5,730 (2,667-6,439) | 7,484 (2,454-11,819) | 5,468 (2,454-7,478) | 5,843 (4,365-9,962) | 5,400 (2,714-9,058) | 5,843 (2,584-10,707) |
| Per child per five years | Mean (SD) | 18,136(26,065) | 16,115 (22,771) | 15,431 (21,891) | 36,200 (38,520) | 22,377 (31,046) | 30,294(38,406) | 22,031(29,641) | 31,514 (39,214) |
|  | Median (IQR) | 9,608(4,867-20,674) | 8,887(4,579-18,713) | 8,668 (4,365-17,855) | 23,849 (11,858-44,475) | 12,373 (5,998-26,347) | 19,362(9,056-35,339) | 12,251 (5,998 – 26,401) | 19,734 (9,133-37,584) |
| Cost per child per year | Mean (SD) | 7,094 (6,505) | 6,554 (5,070) | 6,461 (4,996) | 10,235 (10,792) | 7,997 (8,107) | 6,058(7,681) | 7,401 (6,221) | 9,987 (11,155) |
|  | Median (IQR) | 5412 (3,851-8,454) | 5,234(3,715-82,53) | 5,164 (3,697-8,242) | 7,575 (4,618-11,436) | 6026 (4,042-9,100) | 3,872(1,811-7,068) | 6,001 (4,005-8,882) | 7,104 (4,384-11,068) |

*LBW: Low birthweight*

*PTB: Preterm birth*

*SGA: Small-for-gestational-age*
