# Supplementary material for: Trends in adverse perinatal outcomes and associated hospitalisations, emergency department presentations, and healthcare costs from birth to early childhood in the Northern Territory, Australia: A two-decade population-based study
Source: PLOS Glob Public Health. 2025 Aug 7;5(8):e0004985. doi: 10.1371/journal.pgph.0004985 (PMC12331054; doi:10.1371/journal.pgph.0004985)
Supplement: S2 Table — (DOCX) [file pgph.0004985.s008.docx]

**S2 Table. Proportional mean cost by adverse perinatal outcome in NT, Australia, 2000**–**2020.**

| Adverse perinatal outcome | Mean hospitalisation cost per child per five years | Proportional hospitalisation cost compared to hospitalisation cost of all births | Mean ED presentation cost per child per five years | Proportional ED cost compared to ED cost of all births |
| --- | --- | --- | --- | --- |
| Preterm birth | 30,295 (38,406) | 1.67 | 4,792 (6,078) | 1.33 |
| LBW | 31,515 (39,214) | 1.74 | 4,817 (6,068) | 1.34 |
| SGA | 22,031 (29,641) | 1.21 | 4,083 (5,125) | 1.14 |
| PTB + LBW+ SGA | 36,1999 (38,520) | 2.00 | 5,386 (5,988) | 1.50 |
| Term + Normal birthweight + AGA | 15,431 (21,891) | 0.85 | 3,413 (4,351) | 0.94 |
| At least one outcome (PTB or LBW or SGA) | 22,378 (31,046) | 1.23 | 3,957 (5,045) | 1.10 |
| Total cost of all births | 18,136 (26,065) | 1 (Ref. group) | 3,593 (4,600) | 1(Ref. group) |

*AGA: Appropriate-for-gestational-age*

*LBW: Low birthweight*

*PTB: Preterm birth*

*SGA: Small-for-gestational-age*

The proportional mean cost per child per five years for each adverse outcome was estimated as:

- *Preterm= total cost (mean) of preterm birth divided by total cost (mean) for all births*
- *LBW= total (mean) cost of LBW divided by total (mean) cost for all births*
- *SGA= total (mean) cost of SGA divided by total (mean) cost of all births*
- *All adverse perinatal outcomes= total (mean) cost of all adverse perinatal outcomes (PTB + SGA + LBW) divided by total (mean) cost of all births*
- *At least one adverse perinatal outcome = total (mean) cost of PTB OR SGA OR LBW divided by total (mean) cost of all births*
