# Supplementary material for: Trends in adverse perinatal outcomes and associated hospitalisations, emergency department presentations, and healthcare costs from birth to early childhood in the Northern Territory, Australia: A two-decade population-based study
Source: PLOS Glob Public Health. 2025 Aug 7;5(8):e0004985. doi: 10.1371/journal.pgph.0004985 (PMC12331054; doi:10.1371/journal.pgph.0004985)
Supplement: S3 Table — (DOCX) [file pgph.0004985.s009.docx]

**S3 Table. Cost of hospitalisation by preterm categories from birth to age five years, NT, Australia, 2000**–**2020.**

| **Year of admission** | **Hospitalisation cost per child, median (IQR) (AUD)** | | | |
| --- | --- | --- | --- | --- |
|  | EPTB | VPTB | MPTB | LPTB |
| 2000 | 15,616(4,558-26,860) | 17,214 (10,511- 17,214) | 6,881(4,758- 17,214) | 5,907(3,807- 8,854) |
| 2001 | 14,919 (4,502-26,530) | 10,662 (6,295-17,002) | 9,832 (6,011-17,002) | 4,701 (3,761-7,610) |
| 2002 | 11693 (4,907-27,764) | 7,442 (4,799-17,793) | 7,574 (3,936-10,865) | 6,802 (3,936-9,127) |
| 2003 | 10,954 (6,621-31,156) | 9,374 (7,184-18,792) | 7,715 (4,897-10,867) | 6,825 (4,157-8,468) |
| 2004 | 10,644 (7,325-30,211) | 9,578 (6,868-19,362) | 8,242 (4,494-15,912) | 7,402 (4,282-9,250) |
| 2005 | 8,912 (6,109-17,296) | 11,024 (5,899-19,727) | 8,176 (4,432-12,658) | 7,387 (4,363-9,707) |
| 2006 | 7,898 (5,248-14,083) | 12,101 (8,437-19,819) | 7,577 (4,384-11,113) | 6,086 (4,384-8,437) |
| 2007 | 8,023 (5,237-27,159) | 9,412 (6,476-14,069) | 8,419 (5,028-13,150) | 6,257 (4,375-8,851) |
| 2008 | 5,246 (4,492-19,809) | 8,200 (5,183-18,767) | 7,402 (4,381-11,455) | 5,326 (4,255-8,433) |
| 2009 | 18,607 (9,421-31,631) | 11,236 (6,093-16,719) | 7,750 (4,484-12,378) | 6,164 (4,484-8,630) |
| 2010 | 10,149 (5,985-34,139) | 10,285 (6,938-16,889) | 7,982 (4,618-12,748) | 6,348 (4,553-8,887) |
| 2011 | 17,956 (5,559-45,325) | 12,271 (6,117-20,994) | 8,663 (4,644-13,719) | 6,636 (4,644-9,564) |
| 2012 | 17,449 (5,599-47,886) | 12,886 (5,376-21,147) | 9,256 (5,377-12,912) | 7,395 (4,678-10,103) |
| 2013 | 8.448 (4,689-19,140) | 10,286 (4,780-23,793) | 11,157 (6,460-16,115) | 6,460 (5,008-11,799) |
| 2014 | 7,508 (4,206-27,535) | 8,814 (4,596-27,535) | 9,620 (6,440-14,879) | 8,243 (4,677-12,183) |
| 2015 | 6,745 (4,417-27,102) | 10,910 (5,9934-27,102) | 9,735 (6,783-17,169) | 7,685 (4,437-12,074) |
| 2016 | 7,579 (4,358-29,719) | 8,263 (4,847-15,706) | 9,615 (5,813-18,051) | 7,059 (4,530-9,615) |
| 2017 | 5,361 (3,791-9,961) | 5,645 (3,491-8,775) | 4,791 (2,463-7,478) | 4,849 (2,945-6,765) |
| 2018 | 3,481 (2,727-3,609) | 5,321 (3,404-7,953) | 5,290 (1,849-11,076) | 4,682 (2,289-6,663) |
| 2019 | 4,770 (2,312-5,602) | 8,662 (3,762-11,019) | 5,602 (2,535 -6,806) | 5,511 (3,212-6,473) |
| 2020 | N/A | 7,550 (3,894-11,842) | 7,566(2,196-10,233) | 5,843(4,365- 9,691) |
| Per year | 8,687(5,237- 27,764) | 9,871 (5,700- 19,140) | 8,088(4,677-13,641) | 6,500(4,311-9,620) |
| Per five years | 43,792 (6,522-103,992) | 33,677 (20,878-56,872) | 23286 (14,394-38,280) | 14,541 (7,998-27,712) |

*EPTB: Extreme preterm birth*

*LPTB: Late preterm birth*

*MPTB: Moderate preterm birth*

*VPTB: Very preterm birth*
