# Supplementary material for: Trends in adverse perinatal outcomes and associated hospitalisations, emergency department presentations, and healthcare costs from birth to early childhood in the Northern Territory, Australia: A two-decade population-based study
Source: PLOS Glob Public Health. 2025 Aug 7;5(8):e0004985. doi: 10.1371/journal.pgph.0004985 (PMC12331054; doi:10.1371/journal.pgph.0004985)
Supplement: S4 Table — (DOCX) [file pgph.0004985.s010.docx]

**S4 Table. Hospitalisation cost of PTB by different birthweight and gestational age for birthweight percentiles from birth to age five, NT, Australia, 2000**–**2020.**

| **Year of admission** | | **Mean cost per child ± SD (AUD)** | | | | | |
| --- | --- | --- | --- | --- | --- | --- | --- |
|  |  | **PTB (20 to 36^+6^ weeks of gestational age)** | | | | | |
|  |  | **Small-for-gestational-age (SGA)** | **Appropriate-for-gestational-age (AGA)** | | **Large-for-gestational-age (LGA)** | | |
|  |  | **Birthweight**  **<2,500 grams** | **Birthweight**  **<2,500 grams** | **Birthweight**  **2,500-4,000 grams** | **Birthweight**  **<2,500 grams** | **Birthweight**  **2,500-4,000 grams** | **Birthweight**  **>=4,000 grams** |
| 2000 | | 5,720 (3,982) | 11,068 (14,635) | 8,625 (5,096) | 10,511 (9,480) | 6,557 (2,827) | 10,183 (6,234) |
| 2001 | | 8,832 (10,267) | 9,633 (11,511) | 6,765 (8,882) | 9,870 (6,981) | 7,519 (5,759) | 8,891 (4,239) |
| 2002 | | 9,947 (9,175) | 9,311 (8,836) | 6,905 (4,048) | 8,338 (7,605) | 8,034 (4,831) | 6,057 (3,410) |
| 2003 | | 9,466 (9,262) | 10,192 (12,049) | 8,701 (11,029) | 10,398 (7,599) | 8,777 (10,912) | 10,345 (3,664) |
| 2004 | | 10,084 (12,998) | 11,265 (12,192) | 8,186 (6,317) | 11,082 (14,796) | 7,921 (4,998) | 9,237 (6,150) |
| 2005 | | 10,189 (11,847) | 9,576 (9,679) | 7,772 (4,603) | 16,361 (19,370) | 8,762 (5,281) | 7,588 (5,627) |
| 2006 | | 8,179 (5,077) | 9,919 (10,708) | 7,472 (5,055) | 11,328 (8,691) | 6,693 (4,771) | 7,653 (5,506) |
| 2007 | | 9,687 (9,239) | 9,843 (11,529) | 7,547 (5,270) | 10,354 (6,974) | 8,110 (5,982) | 7,916 (6,151) |
| 2008 | | 10,317 (10,994) | 9,355 (10,506) | 6,555 (4,288) | 8,640 (6,673) | 5,925 (3,129) | 6,046 (2,703) |
| 2009 | | 8,972 (6,419) | 9,936 (10,255) | 7,305 (4,399) | 13,500 (13,538) | 7,631 (5,367) | 7,654 (3,653) |
| 2010 | | 8,845 (5,726) | 10,028 (10,479) | 7,442 (4,784) | 15,052 (17,179) | 8,012 (5,569) | 9,883 (7,285) |
| 2011 | | 10,039 (9,512) | 11,741 (14,666) | 8,055 (9,045) | 12,912 (16,270) | 10,173 (11,356) | 10,416 (9,609) |
| 2012 | | 14,841 (21,324) | 12,435 (15,725) | 7,147 (4,273) | 15,923 (18,164) | 9,780 (5,899) | 8,113 (2,025) |
| 2013 | | 10,448 (9,986) | 12,062 (13,479) | 8,060 (5,942) | 14,315 (21,491) | 8,835 (6,265) | 10,951 (10,172) |
| 2014 | | 10,684 (12,097) | 11,843 (14,478) | 8,957 (13,560) | 14,346 (12,772) | 9,988 (7,937) | 6,443 (2,478) |
| 2015 | | 10,157 (7,525) | 14,442 (20,602) | 7,920 (6,379) | 9,221 (11,067) | 11,405 (14,510) | 8,334 (3,881) |
| 2016 | | 12,208 (19,726) | 12,615 (18,504) | 8,000 (5,627) | 8,957 (6,650) | 9,057 (14,945) | 19,283 (12,971) |
| 2017 | | 5,743 (3,406) | 7,050 (7,013) | 5,535 (4,582) | 5,493 (5,538) | 5,545 (3,973) | 3,467 (1,785) |
| 2018 | | 6,559 (5,699) | 6,096 (5,347) | 5,761 (6,369) | 4,375 (5,38) | 3,917 (2,129) | 2,792 (N/A) |
| 2019 | | 7,595 (6,394) | 6,309 (4,615) | 5,426 (3,435) | 3,958 (2,326) | 6,066 (1,977) | N/A |
| 2020 | | 7,399 (4,109) | 7,575 (4,528) | 5,715 (4,342) | 12,621 (3,579) | 6,459 (4,492) | 5,844 (N/A) |
| Cost per admission (mean) | | 9,855 (11,004) | 10,681 (13,058) | 7,594 (6,947S) | 12,145 (13,698) | 8,464 (8,418) | 9,428 (7,552) |
| Cost per child per five years | Mean (SD) | 36,199 (38,520) | 34,621 (42,676) | 20,644 (27,240) | 46,627 (54,672) | 25,637(30,189) | 26,794 (38,874) |
|  | Median (IQR) | 23,848 (11,858-44,475) | 21,146 (11,196-39,846) | 12,816 (7,184-24,168) | 31,744 (19,529-58,470) | 17,034 (8,026 – 31,276) | 14,802 (8,389 – 23,736) |
| Cost per child per year | Mean (SD) | 7,239 (7,704) | 6,924 (8,535) | 4,129 (5,448) | 9,325 (10,934) | 5,127 (6,037) | 5,358 (7,775) |
|  | Median (IQR) | 4,769 (2,372-8,895) | 4,229 (2,239-7,969) | 2,563 (1,437-4,833) | 6,349 (3,905-11,694) | 3,407 (1,605 – 6,255) | 2,960 (1,677 - 4,747) |

*Low birthweight (<2500 grams)*

*Normal birthweight (2500 to 4000 grams)*

*Overweight (>=4000grams)*

*PTB: Preterm Birth (20 to 36^+6^ weeks of gestational age)*

*Small-for-gestational-age (<10^th^ percentiles of birthweight for gestational age)*

*Appropriate-for-gestational-age (10^th^ to 90^th^ percentiles of birthweight for gestational age)*

*Large-for-gestational-age (>90^th^ percentiles of birthweight for gestational age)*
