# Supplementary material for: Trends in adverse perinatal outcomes and associated hospitalisations, emergency department presentations, and healthcare costs from birth to early childhood in the Northern Territory, Australia: A two-decade population-based study
Source: PLOS Glob Public Health. 2025 Aug 7;5(8):e0004985. doi: 10.1371/journal.pgph.0004985 (PMC12331054; doi:10.1371/journal.pgph.0004985)
Supplement: S5 Table — (DOCX) [file pgph.0004985.s011.docx]

**S5 Table. Hospitalisation cost of term by different birthweight and gestational age for birthweight percentiles from birth to age five, NT, Australia, 2000**–**2020.**

| **Mean cost (AUD) per child ± SD** | | | | | | | | |
| --- | --- | --- | --- | --- | --- | --- | --- | --- |
| **Year of admission** | | **Term births (>=37^+0^ weeks of gestational age)** | | | | | | |
|  |  | **Small-for-gestational-age (SGA)** | | **Appropriate-for-gestational-age (AGA)** | | | **Large-for-gestational-age (LGA)** | |
|  |  | **Birthweight**  **<2,500 grams** | **Birthweight**  **2,500-4,000 grams** | **Birthweight**  **<2,500 grams** | **Birthweight**  **2,500-4,000 grams** | **Birthweight**  **>=4,000 grams** | **Birthweight**  **2,500-4,000 grams** | **Birthweight**  **>=4,000 grams** |
| 2000 | | 5,675 (2,932) | 5,062 (2,636) | 4,381 (1,207) | 5,678 (5,219) | 8,343 (13,795) | 11,058 (11,061) | 5,610 (4,117) |
| 2001 | | 5,646 (3,330) | 6,731 (4,594) | 5,020 (2,660) | 5,824 (3,899) | 5,865 (3,465) | 7,535 (5,771) | 5,694 (3,432) |
| 2002 | | 7,023 (8,137) | 7,204 (4,769) | 4,132 (1,265) | 6,586 (4,978) | 6,469 (7,842) | 7,339 (6,109) | 6,990 (4,515) |
| 2003 | | 8,083 (5,143) | 8,051 (10,114) | 8,786 (3,686) | 6,921 (5,715) | 6,536 (4,454) | 7,679 (4,198) | 6,228 (3,817) |
| 2004 | | 7,679 (4,769) | 8,236 (7,023) | 5,887 (3,044) | 7,208 (6,405) | 6,940 (4,704) | 7,722 (4,786) | 7,588 (4,913) |
| 2005 | | 7,192 (4,540) | 7,651 (5,014) | 6,389 (3,348) | 7,127 (5,819) | 6,371 (5,248) | 7,552 (4,008) | 7,504 (6,714) |
| 2006 | | 7,833 (5,355) | 7,267 (5,277) | 27,770 (55,227) | 6,802 (5,374) | 6,369 (4,030) | 5,469 (2,809) | 6,748 (5,210) |
| 2007 | | 7,416 (4,546) | 7,156 (5,096) | 6,893 (5,186) | 7,136 (5,819) | 7,388 (5,989) | 6,566 (4,392) | 6,768 (5,436) |
| 2008 | | 7,453 (5,429) | 7,428 (6,113) | 6,284 (2,892) | 7,036 (5,803) | 6,023 (4,067) | 8,018 (6,400) | 6,901 (8,642) |
| 2009 | | 7,884 (6,618) | 8,006 (7,347) | 7,603 (4,725) | 7,148 (6,001) | 6,537 (4,581) | 7,035 (4,560) | 6,913 (7,990) |
| 2010 | | 7,986 (5,477) | 8,031 (10,510) | 4,266 (1,631) | 7,123 (6,201) | 6,237 (4,094) | 5,874 (3,375) | 6,351 (3,992) |
| 2011 | | 8,766 (9,618) | 7,611 (4,796) | 6,539 (4,003) | 6,988 (4,728) | 6,240 (4,044) | 6,289 (3,759) | 6,388 (4,319) |
| 2012 | | 8,007 (5,607) | 7,528 (4,883) | 9,536 (7,656) | 7,162 (5,154) | 6,236 (4,208) | 6,764 (3,772) | 6,923 (4,416) |
| 2013 | | 7,702 (5,391) | 6,871 (4,944) | 6,427 (4,402) | 6,905 (6,409) | 6,398 (5,931) | 7,015 (4,545) | 6,381 (4,134) |
| 2014 | | 6,456 (3,998) | 7,496 (13,602) | 13,335 (9,563) | 6,282 (5,627) | 5,568 (3,119) | 5,837 (4,666) | 6,479 (5,296) |
| 2015 | | 7,551 (4,551) | 6,986 (7,409) | 6,694 (2,312) | 6,522 (6,047) | 5,530 (3,662) | 6,123 (4,251) | 7,233 (5,844) |
| 2016 | | 7,242 (5,235) | 6,565 (4,809) | 7,502 (5,949) | 6,633 (5,807) | 5,568 (4,447) | 10,991 (27,825) | 7,034 (5,985) |
| 2017 | | 6,055 (5,563) | 5,915 (6,310) | 4,156 (2,065) | 5,701 (5,715) | 5,142 (4,897) | 6,111 (6,586) | 5,024 (3,626) |
| 2018 | | 8,614 (13,111) | 5,424 (5,402) | 6,472 (4,882) | 5,684 (5,825) | 4,519 (3,177) | 5,034 (2,860) | 5,077 (3,970) |
| 2019 | | 7,492 (6,180) | 6,090 (4,947) | 5,807 (N/A) | 5,784 (6,864) | 8,760 (14,815) | 5,819 (3,865) | 5,729 (4,008) |
| 2020 | | 6,915 (8,532) | 8,126 (8,569) | 4,149 (2,397) | 5,934 (5,112) | 3,338 (1,802) | 6,275 (4,384) | 5,003 (2,089) |
| Cost per admission (mean) | | 7,521 (5,920) | 7,331 (7,025) | 8,440 (15,676) | 6,309 (4,344) | 6,262 (5,272) | 6,985 (8,392) | 6,649 (5,488) |
| Cost per child per five years | Mean(SD) | 22.655 (27,982) | 19,533 (27,892) | 21,250 (33,139) | 15,431 (21,891) | 12,363 (15,073) | 17,093 (25,724) | 14,596 (18,534) |
|  | Median (IQR) | 12,988 (6,430-26,867) | 10,646 (5,083-22,690) | 11,971 (6,992-19,232) | 8,668 (4,365-17,855) | 7,247 (4,220-13,524) | 8,983 (4,994-18,318) | 8,593 (4,484-7,259) |
| Cost per child per year | Mean(SD) | 4,531 (5,596) | 3,907 (5,578) | 4,250(6,628) | 3,086 (4,378) | 2.472 (3,014) | 3,418 (5,145) | 2,919 (3,706) |
|  | Median (IQR) | 2,597 (1,286-5,373) | 2,129 (1,016-4,538) | 2,394 (1,398-3,846) | 1,733 (873-3,571) | 1,449 (844-2,705) | 1,796 (998-3,663) | 1,718 (896-3,451) |

*Low birthweight (<2500 grams)*

*Normal birthweight (2500 to 4000 grams)*

*Overweight (>=4000grams)*

*Small-for-gestational-age (<10^th^ percentiles of birthweight for gestational age)*

*Appropriate-for-gestational-age (10^th^ to 90^th^ percentiles of birthweight for gestational age)*

*Large-for-gestational-age (>90^th^ percentiles of birthweight for gestational age)*
