# Supplementary material for: Trends in adverse perinatal outcomes and associated hospitalisations, emergency department presentations, and healthcare costs from birth to early childhood in the Northern Territory, Australia: A two-decade population-based study
Source: PLOS Glob Public Health. 2025 Aug 7;5(8):e0004985. doi: 10.1371/journal.pgph.0004985 (PMC12331054; doi:10.1371/journal.pgph.0004985)
Supplement: S6 Table — (DOCX) [file pgph.0004985.s012.docx]

**S6 Table. Cost of ED presentation by adverse perinatal outcomes and years from birth to age five years, NT, Australia, 2000**–**2020.**

| **Year of hospitalisation** | | **Cost per child, median (IQR) (AUD)** | | | | | | | |
| --- | --- | --- | --- | --- | --- | --- | --- | --- | --- |
|  |  | **Overall** | **Term** | **Term with no LBW and SGA** | **Preterm + LBW + SGA** | **At least one adverse perinatal outcome** | **PTB** | **SGA** | **LBW** |
| 2000 | | 890 (853-890) | 890 (792-890) | 890 (823-890) | 743 (596-890) | 890 (890-890) | 890 (890-890) | 890 (743-890) | 890 (890-890) |
| 2001 | | 889 (742-889) | 889 (742-889) | 889 (742-889) | 889 (860-889) | 889 (742-889) | 889 (742-889) | 889 (742-889) | 889 (742-889) |
| 2002 | | 742 (590-891) | 730 (582-891) | 698 (573-891) | 891 (631-891) | 764 (597-891) | 803 (597-919) | 847 (597-894) | 853 (597-899) |
| 2003 | | 631 (457-960) | 631 (454-940) | 626 (457-925) | 763 (573-1,241) | 663 (457-1,023) | 760 (524-1,086) | 695 (460-1,037) | 820 (528-1,212) |
| 2004 | | 603 (449-890) | 583 (449-875) | 568 (449-853) | 762 (473-1,061) | 628 (449-953) | 715 (473-1,103) | 630 (454-995) | 750 (496-1,106) |
| 2005 | | 607 (452-881) | 593 (452-853) | 579 (450-830) | 788 (493-1,024) | 631 (452-940) | 692 (457-1,024) | 661 (452-989) | 696 (458-1,037) |
| 2006 | | 606 (451-870) | 606 (451-848) | 606 (451-825) | 658 (456-1,134) | 630(453-940) | 686 (456-1,036) | 656 (456-940) | 694 (456-1,035) |
| 2007 | | 606 (449-882) | 603 (449-859) | 603 (449-858) | 784 (582-1,108) | 628 (449-920) | 670 (458-1,009) | 654 (454-936) | 692 (465-1,030) |
| 2008 | | 605 (450-855) | 595 (450-834) | 586 (447-808) | 621 (412-1,018) | 605 (450-933) | 632 (454-1,035) | 619 (450-938) | 664 (456-1,052) |
| 2009 | | 610 (451-889) | 606 (451-863) | 606 (451-856) | 631 (451-960) | 631 (451-940) | 695 (458-1,037) | 631 (451-941) | 700 (457-1,090) |
| 2010 | | 607 (452-937) | 607 (452-896) | 607 (452-891) | 727 (561-1,038) | 632 (457-976) | 681 (467-1,038) | 667 (457-1,025) | 719 (476-1,038) |
| 2011 | | 598 (450-855) | 582 (450-845) | 577 (450-834) | 715 (533-1,033) | 604 (450-937) | 663 (473-1,033) | 629 (455-937) | 693 (473-1,033) |
| 2012 | | 609 (450-898) | 605 (450-889) | 605 (450-885) | 664 (450-1,106) | 629 (456-938) | 734 (478-1,066) | 629 (450-947) | 719 (474-1,067) |
| 2013 | | 627 (454-934) | 627 (448-912) | 621 (449-899) | 771 (497-1,212) | 634 (454-954) | 691 (472-1,081) | 682 (454-984) | 697 (472-1,078) |
| 2014 | | 617 (454-936) | 607 (450-914) | 608 (453-916) | 727 (527-1,105) | 629 (455-938) | 668 (474-1,034) | 637 (455-946) | 686 (455-1,034) |
| 2015 | | 699 (542-967) | 696 (542-941) | 696 (542-941) | 825 (610-1,159) | 722 (542-988) | 788 (576-1,083) | 721 (542-991) | 771 (558-1,083) |
| 2016 | | 709 (560-922) | 697 (551-913) | 691 (548-901) | 819 (646-1,066) | 724 (560-959) | 751 (570-1,009) | 739 (561-972) | 782 (592-1,066) |
| 2017 | | 698 (553-941) | 698 (553-921) | 698 (553-919) | 864 (633-1,129) | 698 (553-956) | 777 (578-1,066) | 711 (553-997) | 782 (570-1,106) |
| 2018 | | 670 (545-892) | 670 (545-891) | 670 (545-891) | 747 (576-1,009) | 691 (551-919) | 691 (545-964) | 691 (558-952) | 691 (552-992) |
| 2019 | | 683 (556-892) | 679 (556-876) | 677 (556-875) | 800 (513-1,183) | 683 (556-910) | 702 (582-983) | 683 (556-910) | 705 (575-988) |
| 2020 | | 706 (561-919) | 697 (561-918) | 690 (561-885) | 815 (599-990) | 716 (575-943) | 712 (588-919) | 772 (588-993) | 772 (588-943) |
| Per five years | Mean (SD) | 4,514 (5,526) | 3,474 (4,408) | 3,413 (4,351) | 5,386 (5,988) | 3,957 (5,045) | 4,792 (6,078) | 4,083 (5,125) | 4,817 (6,068) |
|  | Median (IQR) | 2,156 (1,074-4,274) | 2,102 (1,044-4,134) | 2,058 (1,032-4,057) | 3,108 (1,609-7,520) | 2,363 (1,187-4,731) | 2,773 (1,298-5,810) | 2,464 (1,216-4,927) | 2,832 (1,328-5,791) |
| Per year | Mean (SD) | 719 (328) | 712 (325) | 709 (323) | 815 (353) | 740 (336) | 787 (348) | 753 (339) | 797 (352) |
|  | Median (IQR) | 634 (472-893) | 630 (466-891) | 629 (464-889) | 760 (533-1,081) | 662 (475-938) | 714 (512-1,033) | 674 (477-956) | 735 (514-1,037) |

*LBW: Low birthweight*

*PTB: Preterm Birth*

*SGA: Small-for-gestational age*
