# Supplementary material for: Trends in adverse perinatal outcomes and associated hospitalisations, emergency department presentations, and healthcare costs from birth to early childhood in the Northern Territory, Australia: A two-decade population-based study
Source: PLOS Glob Public Health. 2025 Aug 7;5(8):e0004985. doi: 10.1371/journal.pgph.0004985 (PMC12331054; doi:10.1371/journal.pgph.0004985)
Supplement: S7 Table — (DOCX) [file pgph.0004985.s013.docx]

**S7 Table. Cost of ED presentation by adverse perinatal outcomes and years from birth to age five years, NT, Australia, 2000**–**2020.**

| **Year of ED visit** | **Cost per child, mean (SD) (AUD)** | | | | | | | | | |
| --- | --- | --- | --- | --- | --- | --- | --- | --- | --- | --- |
|  | **Overall** | **Gestational age** | | | **Birthweight for gestational age percentiles** | | | **Birth weight** | | |
|  |  | **Term** | **PTB** | **Excess cost** | **AGA** | **SGA** | **Excess cost** | **Normal** | **LBW** | **Excess cost** |
| 2000 | 825 (129) | 823 (132) | 845 (108) | 22 | 831 (125) | 803 (147) | -28 | 821 (134) | 846 (107) | 25 |
| 2001 | 804 (136) | 804 (137) | 805 (131) | 1 | 801 (140) | 822 (115) | 21 | 804 (137) | 814 (128) | 10 |
| 2002 | 751 (272) | 747 (272) | 791 (279) | 44 | 746 (273) | 802 (274) | 56 | 750 (274) | 796 (277) | 46 |
| 2003 | 725 (341) | 716 (340) | 809 (344) | 93 | 719 (336) | 779 (364) | 60 | 719 (336) | 850 (376) | 131 |
| 2004 | 689 (331) | 678 (326) | 788 (364) | 110 | 684 (326) | 734 (349) | 50 | 682 (326) | 811 (364) | 129 |
| 2005 | 680 (328) | 672 (324) | 760 (360) | 88 | 672 (327) | 730 (335) | 58 | 675 (324) | 771 (361) | 96 |
| 2006 | 689 (331) | 681 (328) | 758 (353) | 77 | 679 (327) | 735 (337) | 56 | 685 (328) | 757 (343) | 72 |
| 2007 | 688 (332) | 682 (331) | 749 (344) | 67 | 684 (335) | 725 (337) | 41 | 685 (333) | 766 (350) | 81 |
| 2008 | 672 (331) | 665 (326) | 740 (365) | 75 | 667 (328) | 707 (337) | 40 | 666 (325) | 753 (355) | 87 |
| 2009 | 697 (334) | 689 (331) | 778 (357) | 89 | 694 (330) | 730 (352) | 36 | 691 (330) | 787 (366) | 96 |
| 2010 | 707 (340) | 701 (339) | 773 (346) | 72 | 700 (338) | 760 (348) | 60 | 704 (340) | 786 (343) | 82 |
| 2011 | 682 (324) | 674 (319) | 772 (372) | 98 | 677 (321) | 722 (337) | 45 | 676 (319) | 785 (370) | 109 |
| 2012 | 707 (335) | 698 (331) | 796 (360) | 98 | 704 (331) | 730 (360) | 26 | 701 (329) | 794 (373) | 93 |
| 2013 | 721 (342) | 713 (337) | 799 (383) | 86 | 718 (341) | 764 (362) | 46 | 719 (340) | 802 (367) | 83 |
| 2014 | 717 (346) | 711 (345) | 778 (358) | 67 | 716 (345) | 740 (367) | 24 | 718 (348) | 783 (370) | 65 |
| 2015 | 789 (329) | 782 (325) | 855 (361) | 73 | 788 (326) | 795 (339) | 7 | 787 (326) | 849 (371) | 62 |
| 2016 | 772 (297) | 766 (294) | 831 (323) | 65 | 768 (295) | 800 (304) | 32 | 766 (293) | 848 (324) | 82 |
| 2017 | 776 (319) | 770 (318) | 837 (328) | 67 | 774 (309) | 799 (356) | 25 | 772 (315) | 846 (342) | 74 |
| 2018 | 756 (313) | 753 (311) | 786 (334) | 33 | 753 (319) | 772 (300) | 19 | 752 (312) | 798 (344) | 46 |
| 2019 | 756 (319) | 751 (322) | 795 (294) | 44 | 752 (315) | 774 (329) | 22 | 752 (318) | 816 (309) | 64 |
| 2020 | 785 (321) | 784 (324) | 793 (295) | 9 | 772 (315) | 854 (356) | 82 | 780 (323) | 818 (322) | 38 |
| Overall | 719 (328) | 712 (325) | 789 (348) | 77 | 715 (325) | 753 (340) | 38 | 715 (325) | 797 (352) | 82 |

*AGA: Appropriate-for-gestational age*

*ED: Emergency Department*

*LBW: Low birthweight*

*PTB: Preterm Birth*

*SGA: Small-for-gestational age*
