# Supplementary material for: Trends in adverse perinatal outcomes and associated hospitalisations, emergency department presentations, and healthcare costs from birth to early childhood in the Northern Territory, Australia: A two-decade population-based study
Source: PLOS Glob Public Health. 2025 Aug 7;5(8):e0004985. doi: 10.1371/journal.pgph.0004985 (PMC12331054; doi:10.1371/journal.pgph.0004985)
Supplement: S8 Table — (DOCX) [file pgph.0004985.s014.docx]

**S8 Table: Cost of ED presentations by preterm categories from birth to age five years, NT, Australia, 2000**–**2020.**

| **Year of ED presentation** | **Cost of ED presentation per child, mean (SD) (AUD)** | | | |
| --- | --- | --- | --- | --- |
|  | **EPTB** | **VPTB** | **MPTB** | **LPTB** |
| 2000 | 890 | N/A | 890 | 821 (128) |
| 2001 | 840 (98) | 834 (125) | 770 (172) | 809 (123) |
| 2002 | 681 (306) | 867 (205) | 743 (244) | 795 (291) |
| 2003 | 908 (381) | 797 (413) | 751 (379) | 817 (325) |
| 2004 | 1,078 (376) | 836 (335) | 771 (351) | 767 (362) |
| 2005 | 1,105 (492) | 763 (345) | 716 (343) | 746 (346) |
| 2006 | 981 (279) | 773 (372) | 684 (328) | 759 (352) |
| 2007 | 822 (340) | 807 (381) | 790 (337) | 727 (336) |
| 2008 | 889 (428) | 779 (340) | 726 (351) | 729 (369) |
| 2009 | 953 (440) | 859 (370) | 811 (384) | 749 (343) |
| 2010 | 1,097 (305) | 832 (355) | 767 (334) | 752 (341) |
| 2011 | 1,064 (514) | 780 (346) | 814 (353) | 743 (360) |
| 2012 | 1,104 (325) | 795 (382) | 775 (363) | 784 (351) |
| 2013 | 916 (415) | 899 (389) | 823 (354) | 770 (382) |
| 2014 | 791 (670) | 874 (364) | 829 (331) | 753 (356) |
| 2015 | 1,004 (936) | 956 (401) | 859 (350) | 829 (342) |
| 2016 | 975 (383) | 930 (329) | 805 (275) | 810 (322) |
| 2017 | 767 (371) | 887 (323) | 885 (353) | 824 (320) |
| 2018 | 840 (321) | 738 (263) | 873 (415) | 779 (331) |
| 2019 | 1,035 (275) | 835 (304) | 802 (268) | 775 (295) |
| 2020 | 1,352 (N/A) | 776 (293) | 785 (415) | 790 (240) |
| Per year | 955 (406) | 835 (357) | 787 (342) | 772 (342) |
| Per five years | 7,227 (8,368) | 5,744 (6,938) | 4,942 (7,259) | 4,514 (5,526) |

*ED: Emergency Department*

*EPTB: Extreme preterm birth*

*LPTB: Late preterm birth*

*MPTB: Moderate preterm birth*

*VPTB: Very preterm birth*

*N/A: No value for the cell*
