# Supplementary material for: Trends in adverse perinatal outcomes and associated hospitalisations, emergency department presentations, and healthcare costs from birth to early childhood in the Northern Territory, Australia: A two-decade population-based study
Source: PLOS Glob Public Health. 2025 Aug 7;5(8):e0004985. doi: 10.1371/journal.pgph.0004985 (PMC12331054; doi:10.1371/journal.pgph.0004985)
Supplement: S9 Table — (DOCX) [file pgph.0004985.s015.docx]

**S9 Table. Drivers of hospitalisation cost from birth to age five years, NT, Australia, 2000**–**2020.**

| **A. Parametric coefficients** | **Estimate** | **Exp(estimate)** | | **Std. Error** | | **t-value** |
| --- | --- | --- | --- | --- | --- | --- |
| Intercept | 8·054 | 3146·35 | | 0·082 | | 98·37*** |
| Indigenous status of mother |  |  | |  | |  |
| Indigenous | Ref. |  | |  | |  |
| Non-indigenous | -0·427 | 0·652 | | 0·019 | | -22·49*** |
| Remoteness of residence |  |  | |  | |  |
| Rural | Ref. |  | |  | |  |
| Urban | -0·085 | 0·918 | | 0·016 | | -5·16*** |
| Apgar score at 1 minute | -0·013 | 0·987 | | 0·005 | | -2·46* |
| Apgar score at 5 minutes | -0·012 | 0·988 | | 0·009 | | -1·32 |
| Frequency of Antenatal care visits | -0·001 | 0·999 | | 0·002 | | -0·14 |
| Congenital malformation at birth |  |  | |  | |  |
| Not diagnosed | Ref. |  | |  | |  |
| Diagnosed | 0·370 | 1·447 | | 0·060 | | 6·20*** |
| Under investigation | 0·371 | 1·449 | | 0·0445 | | 8·32*** |
| Unknown | 0·065 | 1·067 | | 0·034 | | 1·92 |
| First pregnancy |  |  | |  | |  |
| Yes | Ref. |  | |  | |  |
| No | 0·040 | 1·041 | | 0·018 | | 2·27* |
| Mother’s marital status |  |  | |  | |  |
| Single | Ref. |  | |  | |  |
| Married | 0·115 | 1·122 | | 0·015 | | 7·74*** |
| Others^#^ | 0·225 | 1·252 | | 0·029 | | 7·85*** |
| Outcome following birth hospitalisation |  |  | |  | |  |
| Discharge to usual residence | Ref. |  | |  | |  |
| Transferred to acute care facility | 0·434 | 1·543 | | 0·042 | | 10·29*** |
| Left against advice | 0·148 | 1·160 | | 0·062 | | 2·40* |
| Others | 0·089 | 1·093 | | 0·019 | | 4·64*** |
| Unknown | 0·215 | 1·240 | | 0·081 | | 2·65** |
| Sex at birth |  |  | |  | |  |
| Male | Ref. |  | |  | |  |
| Female | -0·083 | 0·920 | | 0·013 | | -6·38*** |
| Parity | 0·025 | 1·025 | | 0·006 | | 4·01*** |
| Mode of delivery |  |  | |  | |  |
| SVD | Ref. |  | |  | |  |
| Breech | 0·079 | 1·082 | | 0·123 | | 0·65 |
| Forceps | 0·011 | 1·011 | | 0·042 | | 0·26 |
| Ventouse | -0·024 | 0·976 | | 0·030 | | -0·78 |
| CS elective | -0·019 | 0·981 | | 0·022 | | -0·89 |
| CS emergency | 0·042 | 1·043 | | 0·018 | | 2·28* |
| Birthweight for gestational age percentiles |  |  | |  | |  |
| SGA | 0·037 | 1·038 | | 0·026 | | 1·41 |
| AGA | Ref. |  | |  | |  |
| LGA | 0·009 | 1·009 | | 0·033 | | 0·27 |
| Year of birth |  |  | |  | |  |
| 2000 | Ref. |  | |  | |  |
| 2001 | 0·049 | 1·050 | | 0·046 | | 1·06 |
| 2002 | 0·109 | 1·115 | | 0·047 | | 2·31* |
| 2003 | 0·151 | 1·163 | | 0·049 | | 3·07** |
| 2004 | 0·240 | 1·271 | | 0·049 | | 4·88*** |
| 2005 | 0·235 | 1·265 | | 0·048 | | 4·82*** |
| 2006 | 0·253 | 1·288 | | 0·048 | | 5·21*** |
| 2007 | 0·285 | 1·330 | | 0·049 | | 5·82*** |
| 2008 | 0·243 | 1·275 | | 0·048 | | 5·02*** |
| 2009 | 0·328 | 1·388 | | 0·048 | | 6·77*** |
| 2010 | 0·286 | 1·331 | | 0·048 | | 5·93*** |
| 2011 | 0·302 | 1·352 | | 0·048 | | 6·23*** |
| 2012 | 0·334 | 1·396 | | 0·048 | | 6·93*** |
| 2013 | 0·350 | 1·419 | | 0·048 | | 7·24*** |
| 2014 | 0·352 | 1·422 | | 0·047 | | 7·35*** |
| 2015 | 0·403 | 1·496 | | 0·048 | | 8·39*** |
| 2016 | 0·394 | 1·483 | | 0·056 | | 7·11*** |
| Mother’s country of birth |  |  | |  | |  |
| Australia | Ref. |  | |  | |  |
| Others | -0·0107 | 0·980 | | 0·022 | | -0·480 |
| **B. Smoother terms** | **edf** | | **Ref. df** | | **F-value** | |
| s(Age of mother’s) | 2·55 | | 3·23 | | 25·64*** | |
| s(Gestational age, in weeks) | 2·76 | | 3·46 | | 0·51 | |
| s(Birthweight, in grams) | 1·39 | | 1·71 | | 1·03 | |
| s(Length of stay following birth hospitalisation, in days) | 4·33 | | 9·00 | | 13·36*** | |
| s(Length of stay following subsequent admission, in days) | 8·89 | | 9·00 | | 298·45*** | |
| ti(Birthweight, Gestational age) | 6·48 | | 7·75 | | 7·25*** | |

*Exp: exponentiated value of the coefficient*

*Statistically significant level *** < 0·001, ** < 0·01, *< 0·05*

*AGA: Appropriate-for-gestational age*

*CS: Caesarean Section*

*LGA: Large-for-gestational age*

*SGA: small-for-gestational age*

*SVD: Spontaneous vaginal delivery*

*edf: Estimated degree of freedom*

*Ref. df: Reference degree of freedom*

*ti(Birthweight, Gestational age): is for tensor interaction term for birthweight and gestational age*

*S: smoother terms*
