# Supplementary material for: Trends in adverse perinatal outcomes and associated hospitalisations, emergency department presentations, and healthcare costs from birth to early childhood in the Northern Territory, Australia: A two-decade population-based study
Source: PLOS Glob Public Health. 2025 Aug 7;5(8):e0004985. doi: 10.1371/journal.pgph.0004985 (PMC12331054; doi:10.1371/journal.pgph.0004985)
Supplement: S10 Table — (DOCX) [file pgph.0004985.s016.docx]

**S10 Table. Percentage change of non-linear co-variates and hospitalisation cost from birth to age five years, NT, Australia, 2000**–**2020.**

| **Reference point for smoother terms** | **% change** |
| --- | --- |
| Age of mother (years) |  |
| 20 | 35.0 |
| 25 | 16.2 |
| 30 | Ref. |
| 35 | -10 |
| Gestational age (weeks) |  |
| 28 | 64.8 |
| 32 | 34.9 |
| 34 | 16 |
| 37 | 5.0 |
| 40 | Ref. |
| Birthweight (grams) |  |
| 1000 | 22.1 |
| 2000 | 10.5 |
| 3000 | 5.1 |
| 3500 | Ref. |
| 4000 | 5.0 |
| 5000 | 10.5 |
| Birth hospitalisation length of stay (days) |  |
| 0 | Ref. |
| 1 | 5.0 |
| 5 | 16.2 |
| 10 | 34.9 |
| 20 | 82.0 |
| 40 | 171.0 |
| Readmission length of stay (days) |  |
| 0 | Ref. |
| 1 | 10.6 |
| 5 | 49.0 |
| 10 | 42.0 |
| 20 | 22.1 |
| 30 | 10.5 |
