# Supplementary material for: Trends in adverse perinatal outcomes and associated hospitalisations, emergency department presentations, and healthcare costs from birth to early childhood in the Northern Territory, Australia: A two-decade population-based study
Source: PLOS Glob Public Health. 2025 Aug 7;5(8):e0004985. doi: 10.1371/journal.pgph.0004985 (PMC12331054; doi:10.1371/journal.pgph.0004985)
Supplement: S11 Table — (DOCX) [file pgph.0004985.s017.docx]

**S11 Table. Drivers of cost of ED presentation from birth to age five years, NT, Australia, 2000**–**2020.**

| **A. Parametric coefficients** | **Coefficient** | **Exp*** | **Std. Error** | **t-value** |
| --- | --- | --- | --- | --- |
| Intercept | 6.7264 | 834.139 | 0.028 | 240.56*** |
| Indigenous status of baby |  |  |  |  |
| Indigenous | Ref. |  |  |  |
| Non-indigenous | -0.097 | 0.907 | 0.005 | -18.04*** |
| Remoteness of residence |  |  |  |  |
| Rural | Ref. |  |  |  |
| Urban | -0.052 | 0.949 | 0.005 | -10.66*** |
| Apgar score at 1 minute | 0.003 | 1.003 | 0.002 | 1.55 |
| Apgar score at 5 minutes | -0.013 | 0.987 | 0.003 | -4.19*** |
| Antenatal care visits | -0.001 | 0.998 | 0.001 | -2.62** |
| Congenital malformation at birth |  |  |  |  |
| Not diagnosed | Ref. |  |  |  |
| Diagnosed | 0.047 | 1.048 | 0.021 | 2.27* |
| Under investigation | 0.056 | 1.057 | 0.015 | 3.66*** |
| Unknown | 0.038 | 1.039 | 0.011 | 3.49*** |
| First pregnancy |  |  |  |  |
| Yes | Ref. |  |  |  |
| No | 0.023 | 1.023 | 0.005 | 4.22*** |
| Mother’s marital status |  |  |  |  |
| Single | Ref. |  |  |  |
| Married | 0.014 | 1.014 | 0.005 | 2.90** |
| Others | 0.017 | 1.017 | 0.008 | 2.14* |
| Unknown | 0.028 | 1.028 | 0.025 | 1.13 |
| Outcome of admission following birth |  |  |  |  |
| Discharge to usual residence | Ref. |  |  |  |
| Transferred to an(other) acute care facility | -0.001 | 0.999 | 0.015 | 0.96 |
| Left against advice | 0.094 | 1.098 | 0.021 | 4.52*** |
| Other | 0.059 | 1.061 | 0.007 | 8.55*** |
| Unknown | 0.053 | 1.054 | 0.027 | 1.92 |
| Sex at birth |  |  |  |  |
| Male | Ref. |  |  |  |
| Female | -0.018 | 0.983 | 0.004 | -4.42*** |
| Parity | 0.015 | 1.015 | 0.002 | 7.76*** |
| Mode of delivery |  |  |  |  |
| SVD | Ref. |  |  |  |
| Forceps | -0.021 | 0.979 | 0.013 | -1.66 |
| Ventouse | 0.001 | 1.001 | 0.009 | 0.08 |
| CS elective | -0.008 | 0.991 | 0.006 | -1.32 |
| CS emergency | -0.012 | 0.988 | 0.006 | -2.01 |
| Birthweight for gestational age percentiles |  |  |  |  |
| SGA | -0.009 | 0.991 | 0.009 | -0.98 |
| AGA | Ref. |  |  |  |
| LGA | 0.0044 | 1.004 | 0.011 | 0.40 |
| Year of birth |  |  |  |  |
| 2000 | Ref. |  |  |  |
| 2001 | -0.051 | 0.950 | 0.016 | -3.25** |
| 2002 | -0.072 | 0.930 | 0.016 | -4.48*** |
| 2003 | -0.064 | 0.938 | 0.016 | -3.89*** |
| 2004 | -0.040 | 0.960 | 0.017 | -2.42* |
| 2005 | -0.031 | 0.969 | 0.016 | -1.91 |
| 2006 | -0.031 | 0.968 | 0.016 | -1.93 |
| 2007 | -0.036 | 0.964 | 0.016 | -2.23* |
| 2008 | -0.025 | 0.975 | 0.016 | -1.57 |
| 2009 | -0.016 | 0.984 | 0.016 | -0.98 |
| 2010 | 0.007 | 1.007 | 0.016 | 0.43 |
| 2011 | 0.006 | 1.006 | 0.016 | 0.38 |
| 2012 | 0.030 | 1.031 | 0.016 | 1.91 |
| 2013 | 0.095 | 1.100 | 0.015 | 6.00*** |
| 2014 | 0.128 | 1.136 | 0.015 | 8.01*** |
| 2015 | 0.148 | 1.159 | 0.015 | 9.29 *** |
| 2016 | 0.165 | 1.179 | 0.019 | 9.23*** |
| **B. Smoother terms** | **edf** | | **Ref. df** | **F-value** |
| s(Age of mother’s) | 3.82 | | 4.765 | 23.12*** |
| s(Gestational age, in weeks) | 1.62 | | 2.041 | 2.77 |
| s(Birthweight, in grams) | 4.93 | | 6.067 | 3.73** |
| s(Length of stay following birth hospitalisation, in days) | 2.62 | | 9.00 | 6.26*** |
| s(Length of ED stay, in hours) | 4.31 | | 9.00 | 5.74*** |
| ti(birthweight, gestational age) | 3.52 | | 4.337 | 2.48* |

*Exp: exponentiated value of the coefficient*

*Statistically significant level *** < 0.001, ** < 0.01, *< 0.05*

*AGA: Appropriate-for-gestational-age*

*CS: Caesarean Section*

*ED: Emergency Department,*

*LGA: Large-for-gestational-age*

*SGA: small-for-gestational-age*

*SVD: Spontaneous vaginal delivery*

*EDF: Estimated degree of freedom for the smoother terms*

*Ref. df: Reference degree of freedom*

*ti(Birthweight, Gestational age): is for tensor interaction term for birthweight and gestational age.*

*S: Smoother term*
