# Supplementary material for: Trends in adverse perinatal outcomes and associated hospitalisations, emergency department presentations, and healthcare costs from birth to early childhood in the Northern Territory, Australia: A two-decade population-based study
Source: PLOS Glob Public Health. 2025 Aug 7;5(8):e0004985. doi: 10.1371/journal.pgph.0004985 (PMC12331054; doi:10.1371/journal.pgph.0004985)
Supplement: S12 Table — (DOCX) [file pgph.0004985.s018.docx]

**S12 Table. Percentage change of non-linear co-variates and cost of ED presentations from birth to age five years, NT, Australia, 2000**–**2020.**

| **Variables** | **% Change** |
| --- | --- |
| Age of mother (years) |  |
| 20 | 10.0 |
| 25 | 5.0 |
| 30 | Ref. |
| 35 | 3.0 |
| Gestational age (weeks) |  |
| 28 | 20.0 |
| 32 | 16.0 |
| 34 | 10.0 |
| 37 | 7.0 |
| 40 | Ref. |
| Birthweight (grams) |  |
| 1000 | 16.0 |
| 2000 | 12.0 |
| 3000 | 7.0 |
| 3500 | Ref. |
| 4000 | 7.0 |
| 5000 | 9.0 |
| Birth hospitalisation length of stay (days) |  |
| 0 | Ref. |
| 1 | 10.0 |
| 5 | 11.0 |
| 10 | 14.0 |
| 20 | 18.0 |
| 40 | 21.0 |
| ED stay (hours) |  |
| 0 | Ref. |
| 1 | 1.0 |
| 5 | 5.0 |
| 10 | 8.0 |
| 20 | 10.0 |
| 30 | 4.0 |
