# Supplementary material for: Trends in adverse perinatal outcomes and associated hospitalisations, emergency department presentations, and healthcare costs from birth to early childhood in the Northern Territory, Australia: A two-decade population-based study
Source: PLOS Glob Public Health. 2025 Aug 7;5(8):e0004985. doi: 10.1371/journal.pgph.0004985 (PMC12331054; doi:10.1371/journal.pgph.0004985)
Supplement: S13 Table — (DOCX) [file pgph.0004985.s019.docx]

**S13 Table. Drivers of cost of hospitalisation and ED presentation from birth to age five years, NT, Australia, 2000**–**2020.**

| **A. Parametric coefficients** | **Estimate** | **Std. Error** | **t-value** |
| --- | --- | --- | --- |
| Intercept | 8.95 | 0·04 | 199*** |
| Age of mother, years | -0.002 | 0.0005 | -4.03^***^ |
| Birthweight, grams | 0.00001 | 0.000007 | 1.62 |
| Indigenous status of baby |  |  |  |
| Indigenous | Ref. |  |  |
| Non-indigenous | -0·051 | 0·082 | -6.23*** |
| Remoteness of residence |  |  |  |
| Rural | Ref. |  |  |
| Urban | -0.022 | 0·007 | -3.08** |
| Apgar score at 1 minute | 0·003 | 0·002 | -1·45 |
| Apgar score at 5 minutes | -0·015 | 0·004 | -3.61*** |
| Antenatal care visits | -0·0005 | 0·0006 | -0.81 |
| Congenital malformation at birth |  |  |  |
| Not diagnosed | Ref. |  |  |
| Diagnosed | 0·114 | 0·027 | 4.27*** |
| Under investigation | 0·085 | 0·015 | 4.19*** |
| Unknown | -0·002 | 0·015 | -1.63 |
| First pregnancy |  |  |  |
| Yes | Ref. |  |  |
| No | 0·006 | 0·007 | 0.82 |
| Mother’s marital status |  |  |  |
| Single | Ref. |  |  |
| Married | 0·014 | 0·005 | 2·90** |
| Others | 0·017 | 0·008 | 2·14* |
| Unknown | 0·028 | 0·025 | 1·13 |
| Outcome of admission following birth |  |  |  |
| Discharge to usual residence | Ref. |  |  |
| Transferred to an(other) acute care facility | -0·044 | 0·018 | -2.31^*^ |
| Left against advice | 0·068 | 0·027 | 2.53* |
| Other | 0·018 | 0·008 | 2.09* |
| Unknown | -0·026 | 0·036 | -0.73 |
| Parity | 0·008 | 0·003 | 3.13** |
| Year of birth |  |  |  |
| 2000 | Ref. |  |  |
| 2001 | 0·030 | 0·021 | 1.46 |
| 2002 | 0·067 | 0·021 | 3.14** |
| 2003 | 0·083 | 0·022 | 3.75*** |
| 2004 | 0·115 | 0·022 | 5.20*** |
| 2005 | 0·113 | 0·022 | 5.10*** |
| 2006 | 0·118 | 0·022 | 5.34*** |
| 2007 | 0·132 | 0·022 | 6.00*** |
| 2008 | 0·146 | 0·022 | 7.63*** |
| 2009 | 0·191 | 0·021 | 8.73*** |
| 2010 | 0·195 | 0·021 | 8.93*** |
| 2011 | 0·194 | 0·021 | 8.85*** |
| 2012 | 0·198 | 0·021 | 9.08*** |
| 2013 | 0·182 | 0·021 | 8.34*** |
| 2014 | 0·225 | 0·021 | 10.35*** |
| 2015 | 0·235 | 0·021 | 10.81*** |
| 2016 | 0·301 | 0·024 | 12.54*** |
| Country of birth, for mothers |  |  |  |
| Australia | Ref. |  |  |
| Others | 0.028 | 0.01 | 2.84** |
| **B. Smoother terms** | **edf** | **Ref. df** | **F-value** |
| s(Gestational age, in weeks) | 2.86 | 3.59 | 1.36 |
| s(Length of stay following birth hospitalisation, in days) | 3.00 | 9·00 | 3.35*** |
| s(Length of ED stay, in hours) | 3.38 | 9·00 | 1.30** |
| S(length of stay following readmission, days) | 8.57 | 9.00 | 695*** |
| ti(birthweight, gestational age): AGA | 5.78 | 7.00 | 5.35*** |
| ti(birthweight, gestational age): SGA | 4.49 | 5.91 | 13.16*** |
| ti(birthweight, gestational age): LGA | 2.14 | 2.74 | 4.92** |

*Exp: exponentiated value of the coefficient*

*Statistically significant level *** < 0·001, ** < 0·01, *< 0·05*

*AGA: Appropriate-for-gestational age*

*CS: Caesarean Section*

*ED: Emergency Department*

*LGA: Large-for-gestational age*

*SGA: small-for-gestational age*

*SVD: Spontaneous vaginal delivery*

*EDF: Estimated degree of freedom for the smoother terms*

*Ref. df: Reference degree of freedom*

*ti(birthweight, gestational age): is for tensor interaction for birthweight and gestational age*
