# Supplementary material for: Trends in adverse perinatal outcomes and associated hospitalisations, emergency department presentations, and healthcare costs from birth to early childhood in the Northern Territory, Australia: A two-decade population-based study
Source: PLOS Glob Public Health. 2025 Aug 7;5(8):e0004985. doi: 10.1371/journal.pgph.0004985 (PMC12331054; doi:10.1371/journal.pgph.0004985)
Supplement: Attachment — Submitted filename: PGPH-D-25-00279_reviewer.pdf [file pgph.0004985.s020.pdf]

PLOS Global Public Health

Trends in adverse perinatal outcomes and associated hospitalisations, emergency department presentations, and healthcare costs from birth to early childhood in Northern Territory, Australia: A two-decade population-based study

--Manuscript Draft--

Manuscript Number:	PGPH-D-25-00279
Article Type:	Research Article
Full Title:	Trends in adverse perinatal outcomes and associated hospitalisations, emergency department presentations, and healthcare costs from birth to early childhood in Northern Territory, Australia: A two-decade population-based study
Short Title:	Trends in adverse perinatal outcomes and associated health and economic burdens
Corresponding Author:	Tsegaye G. Haile<br>Curtin University<br>Perth, WA AUSTRALIA
Order of Authors:	Tsegaye G. Haile
	Gavin Pereira
	Richard Norman
	Gizachew A. Tessema
Keywords:	Adverse perinatal outcomes; preterm birth; small-for-gestational-age; low birthweight; trends; hospitalisation; ED presentation; economic burden; early childhood; GAM
Abstract:	Adverse perinatal outcomes, including preterm birth (PTB), small-for-gestational-age (SGA), and low birthweight (LBW), are major contributors to childhood health challenges and healthcare demands. We investigated trends in PTB, SGA, and LBW, as well as associated hospitalisations, emergency department (ED) presentations, and healthcare costs in the first five years of age, from a health system perspective in the Northern Territory, Australia. We conducted a retrospective cohort study of births from July 1, 2000, to June 30, 2016, using the Perinatal Trends dataset linked to Inpatient and ED activity (July 1, 2000, to June 30, 2021), and cost-weight data. Healthcare costs, standardised to June 2024 Australian Dollars (AUD), included direct medical and non-medical costs. A Generalized Additive Model with a gamma distribution and a log link function was used to identify cost drivers. We linked 31,183 births to hospitalisation and 42,174 births to ED presentation records. The incidence of PTB increased (81 to 87 per 1,000 live births), while SGA declined (152 to 113). Mean hospitalisations increased for PTB (1.3 ± 0.7 to 6.9 ± 6.0), LBW (1.3 ± 0.6 to 7.2 ± 5.2), and SGA (1.2 ± 0.6 to 8.1 ± 15.1), while readmission durations decreased. Mean ED presentations increased for PTB (1.3 ± 0.5 to 11.5 ± 10.7), SGA (2.2 ± 1.9 to 12.2 ± 11.5), and LBW (1.2 ± 0.2 to 10.9 ± 8.7). The median five-year hospitalisation cost for a PTB child with LBW and SGA was AUD 23,848 (IQR: 11,858–44,475), compared to AUD 8,668 (IQR: 4,365–17,855) for a term child without these outcomes. Median ED costs were AUD 3,108 (IQR: 1,609–7,520) for a PTB child with LBW and SGA and AUD 2,058 (IQR: 1,032–4,057) for a term child without LBW and SGA. Median hospitalisation cost increased for SGA (AUD 3,807 to AUD 5,245) and LBW (AUD 5,234 to AUD 5,843) but decreased for PTB (AUD 6,581 to AUD 6,219). Hospitalisation, ED presentations, and costs were higher in later cohorts, Indigenous children, those in remote areas, and for those with prolonged hospital stay, though individual costs declined with child age. The burden of adverse perinatal outcomes increased in later cohorts, resulting in higher hospitalisation, ED presentation and costs, particularly among Indigenous and remote children, as well as those born with extreme gestational age or birthweight. Costs were primarily incurred through extended hospital stays. Future studies should quantify the burdens across different population groups to help better informed policy.
Additional Information:	
**Question**	**Response**
Financial Disclosure	This research was supported by the Australian National Health and Medical Research

Powered by Editorial Manager® and ProduXion Manager® from Aries Systems Corporation

Enter a financial disclosure statement that describes the sources of funding for the work included in this submission and the role the funder(s) played. This includes grants and any commercial funding of the work or authors.<br><br>This statement will be typeset if the manuscript is accepted for publication.<br><br>*Please review the submission guidelines and the instructions link below for detailed requirements and guidance.*	Council (NHMRC) Investigator Grant (#1195716) to GAT, and NHMRC Project (#1099655) and investigator grant (#1173991) GP. TGH is supported by Research Training Programme linked to NHMRC.
**Competing Interests**<br><br>On behalf of all authors, disclose any competing interests that could be perceived to bias this work.<br><br>This statement will be typeset if the manuscript is accepted for publication.<br><br>*Please review the instructions link below and PLOS Global Public Health's competing interests policy to determine what information must be disclosed at submission.*	The authors have declared that no competing interests exist.
**Data Availability**<br><br>Before publication, Authors are required to make fully available and without restriction all data underlying their findings. Please see our PLOS Data Policy page for detailed information on this policy.<br><br>A **Data Availability Statement**, detailing where the data can be accessed, is required at first submission. Insert your Data Availability Statement in the box	The dataset is the property of the Northern Territory Department of Health, Australia, and is not public available. Access to the datasets should be upon request and approval of ethics committee and data custodians and requests can be directed to SA-NT data link (https://www.santdatalink.org.au/).

below.<br><br>Please see the data reporting section of our submission guidelines for instructions on what you need to include in your Data Availability Statement.<br><br>This statement will be typeset if the manuscript is accepted for publication.<br><br>*PLOS allows rare exemptions to address legal and ethical concerns. If you have legal or ethical restrictions, please use the box below to detail these in full sentences for the Journal team to consider.*	

Powered by Editorial Manager® and ProduXion Manager® from Aries Systems Corporation

Manuscript

Click here to access/download;Manuscript;Manuscript_Haile et al..docx

Trends in adverse perinatal outcomes and associated hospitalisations, emergency department presentations, and healthcare costs from birth to early childhood in Northern Territory, Australia: A two-decade population-based study

Tsegaye G. Haile[1,2*], Gavin Pereira[1,3,4], Richard Norman[1,3], Gizachew A. Tessema[1,3,5]

[1]Curtin School of Population Health, Curtin University, WA, Australia

[2]Department of Health Systems and Policy, Institute of Public Health, University of Gondar, Ethiopia

[3]enAble Institute, Curtin University, WA, Australia

[4]WHO Collaborating Centre for Climate Change and Health Impact Assessment. Faculty of Health Science, Curtin University, WA, Australia

[5]School of Public Health, University of Adelaide, SA, Australia

[*]Corresponding author

Tsegaye G. Haile, Curtin School of Population Health, Curtin University, WA, Australia 6102

Email: tsegaye.haile@curtin.edu.au

Abstract

Adverse perinatal outcomes, including preterm birth (PTB), small-for-gestational-age (SGA), and low birthweight (LBW), are major contributors to childhood health challenges and healthcare demands. We investigated trends in PTB, SGA, and LBW, as well as associated hospitalisations, emergency department (ED) presentations, and healthcare costs in the first five years of age, from a health system perspective in the Northern Territory, Australia. We conducted a retrospective cohort study of births from July 1, 2000, to June 30, 2016, using the Perinatal Trends dataset linked to Inpatient and ED activity (July 1, 2000, to June 30, 2021), and cost-weight data. Healthcare costs, standardised to June 2024 Australian Dollars (AUD), included direct medical and non-medical costs. A Generalized Additive Model with a gamma distribution and a log link function was used to identify cost drivers. We linked 31,183 births to hospitalisation and 42,174 births to ED presentation records. The incidence of PTB increased (81 to 87 per 1,000 live births), while SGA declined (152 to 113). Mean hospitalisations increased for PTB (1.3 ± 0.7 to 6.9 ± 6.0), LBW (1.3 ± 0.6 to 7.2 ± 5.2), and SGA (1.2 ± 0.6 to 8.1 ± 15.1), while readmission durations decreased. Mean ED presentations increased for PTB (1.3 ± 0.5 to 11.5 ± 10.7), SGA (2.2 ± 1.9 to 12.2 ± 11.5), and LBW (1.2 ± 0.2 to 10.9 ± 8.7). The median five-year hospitalisation cost for a PTB child with LBW and SGA was AUD 23,848 (IQR: 11,858–44,475), compared to AUD 8,668 (IQR: 4,365–17,855) for a term child without these outcomes. Median ED costs were AUD 3,108 (IQR: 1,609–7,520) for a PTB child with LBW and SGA and AUD 2,058 (IQR: 1,032–4,057) for a term child without LBW and SGA. Median hospitalisation cost increased for SGA (AUD 3,807 to AUD 5,245) and LBW (AUD 5,234 to AUD 5,843) but decreased for PTB (AUD 6,581 to AUD 6,219). Hospitalisation, ED presentations, and costs were higher in later cohorts, Indigenous children, those in remote areas, and for those with prolonged hospital stay, though individual costs declined with child age. The burden of adverse perinatal outcomes increased in later cohorts, resulting in higher hospitalisation, ED presentation and costs, particularly among Indigenous and remote children, as well as those born with extreme gestational age or birthweight. Costs were primarily incurred through extended hospital stays. Future studies should quantify the burdens across different population groups to help better informed policy.

Introduction

Despite global health advancements, perinatal morbidity such as preterm birth (PTB)[1, 2], small-for-gestational-age (SGA), and low birthweight (LBW)[3], as well as stillbirth[4] and neonatal mortality, continue to impose a substantial dual burden of clinical and economic strain, with far-reaching short- and long-term consequences for individuals, healthcare systems, and society.

The prevalence, short- and long-term complications of perinatal morbidity and mortality, and health management, have been well documented in high-income countries such as Australia[5-7]. Accordingly, the trends and burden of perinatal morbidity and mortality have shown no significant decrease over time. As reported by the Global Burden of Disease 2021, PTB remains the leading cause of under-five mortality, followed by encephalopathy, congenital heart defects, and other disorders[5]. In 2021, the Australian Institute of Health and Welfare reported 9.6 perinatal deaths per 1,000 total births, 75% of which are stillbirths[8]. In the Northern Territory (NT), Australia, a region characterised greater relative socioeconomic disadvantage, this burden was more than double, with 20.6 perinatal deaths per 1,000 total births. Additionally, the region experiences elevated rates of PTB[5] and other perinatal morbidities, necessitating coordinated efforts to reduce the burden and meet national target levels. Due to the advancement of care and management the survival and outcomes of children born with adverse outcomes has been improved. Similarly, their length and frequency of health facility visit has increased[7, 9, 10]. These improvements might have profound economic burden to the healthcare system though more intensive resource use.

Only a small number of studies have measured the cost associated with PTB over limited time periods in Australia. In one example, a study by Callander et al.[11] examined the two-year cost of preterm and extremely preterm infants in Queensland, Australia, finding that the mean annual hospitalisation cost was $182,312 for extremely preterm babies (22 to 28 weeks gestation) in the first year and $9,958 in the second year. Another study by Stephens et al.[7] in New South Wales, Australia, assessed the hospitalisation and acute care costs of very and moderately preterm infants over the first six years of life, reported particularly high cost in the first year following birth. These studies focused solely on PTB and were limited to a few cohort years, covering both births and follow-up for a small number of years, making it difficult to capture how costs change with advancements in healthcare and efforts to meet national targets over time. Furthermore, they were limited to individual states, meaning we have a scarcity of evidence from the NT examining the health and economic burden of a border range of adverse perinatal outcomes[12]. The NT is one of the Australia’s most remote and sparsely populated region with an estimated population of 232,605 according to the 2021 Population and Housing Census[13, 14]. It has a higher proportion of Indigenous residents compared to other Australian states, and higher proportion of health burden has been reported in the region.

Children born with adverse perinatal outcome(s) experience longer hospitalisation, more frequent visits, and a higher level of intensive care; consequently, leads to substantial healthcare resource use, resulting in a significant short- and long-term economic impact on the healthcare system. Therefore, in our study, we aimed to i) estimate the incidence and trends of adverse perinatal outcomes (PTB, SGA, and LBW); ii) measure hospitalisation and ED presentation rates and the trends; and iii) analyse associated healthcare costs during the first five years of life over the last two decades and identifying the drivers of these costs from a health system perspective in NT, Australia.

Examining the economic burden trends for children born with adverse perinatal outcomes throughout childhood and across populations can support efforts to enhance perinatal health interventions by prioritising resource allocation and informing the design of more effective, cost-efficient health approaches by providing cost-related information. Our results also provide a basis for estimating potential savings from programs aimed at reducing adverse perinatal incidence or improving perinatal health outcomes.

Methods

Study design and data linkage

We conducted a population-based retrospective cohort study using data on births from July 01, 2000, to June 30, 2016, in the NT, Australia. Administrative data were obtained by linking records from the Perinatal Trends dataset with the Inpatient and Emergency Department (ED) activity datasets. The Inpatient and ED activity datasets covered the period from July 1, 2000, to June 30, 2021. Additionally, cost-weight data were obtained from the National Hospital Cost Data Collection (NHCDC), specifically the Australian Refined Diagnosis Related Groups (AR-DRGs) for inpatient services, and Urgency Related Groups (URG) for ED costs, available from the Independent Health and Aged Care Pricing Authority[15].

The AR-DRGs provide a clinically meaningful way to relate or group the number and type of patients treated in an admission of care to the resources required in treatment and patients with similar AR-DRGs group are requiring similar hospital services. This classification has been updated every three years along with the International Statistical Classification of Diseases and Related Health Problems, Australian Classification of Health Interventions, Australian Coding Standards classification. Similarly, the URG classify patients presented to ED and group them based on their similar urgency services.

Study population and cohort

Our study included all singleton live births from July 1, 2000, to June 30, 2016. Adverse perinatal outcomes in our study included PTB, SGA, and LBW. PTB, births occurring before 37 completed weeks of gestational age, is further classified as extreme PTB ($<27^{+6}$ weeks), very PTB (28^{+0} to 31^{+6} weeks), moderate PTB (32^{+0} to 33^{+6} weeks), and late PTB (34^{+0} to 36^{+6} weeks)[16]. We classified the birthweight

by gestational age for singleton births using the national birthweight percentiles by gestational age and classified them as SGA (<10th percentiles), appropriate-for-gestational-age (AGA) (10th to 90th percentiles), and larger-for-gestational-age (LGA) (>90th percentiles)[17]. LBW was defined as a birthweight of less than 2,500 grams.

Cost estimations and adjustment

In our study, we used an incidence-based approach of cost estimation which will highlight the likely savings from perinatal health programmes through reducing perinatal morbidity incidence or improvement of their health outcomes[18]. Accordingly, we examined the costs (cost of hospitalisation and ED presentation) related to PTB, LBW, and SGA. We estimated the cost from a health system perspective, including both direct and overhead costs. Direct costs refer to expenses directly related to health services, while overhead costs, which refer to indirect costs, include facility and utility services.

The cost weight per AR-DRG and URG from 2012 onwards are publicly available[15]. To estimate the cost weight per AR-DRG and URG for the period between 2000 and 2012, we deflated the 2012 value using the national medical services related consumer price index (CPI)[19]. Then we adjusted and reported all the cost to June 2024 value, in Australian Dollars (AUD), using the NT specific health related CPI. Finally, we estimated the incremental cost associated with adverse perinatal outcomes and the changes over time by calculating the difference in costs compared to those who presented to inpatient and ED services without adverse perinatal outcomes from birth to age five years.

Inclusion criteria

Our study cohort (all singleton live births from July 1, 2000, to June 30, 2016) was selected to ensure a completed follow-up of five years, as of June 30, 2021, to capture cost of hospitalisation and ED presentation incurred during the first five years of life.

Observations with a birthweight below 400 grams and a gestational age less than 20 weeks were excluded as non-viable births. The child age was calculated using the date of admission and date of birth, and the first day of the month was assigned as the birth date because the perinatal dataset only provided the month and year of birth (**Fig 1**).

Fig 1. Cohort selection flow diagram, NT, Australia, 2000 to 2016.

ED: Emergency Department, IP: Inpatient admission, GA: Gestational age (in weeks), BWt: Birthweight (in grams),
These cohorts were had a minimum of five years as of June 30, 2021, and selected to capture the hospitalisation/ED presentation and the associated costs in the first five years of life.
**These children included in the final analysis were had at least one admission (for IP admission), or one ED presentation (for ED presentation), or one admission and ED presentation (for combined) in the first five years of life.*

Statistical analysis

First, we conducted descriptive analyses, including frequencies and percentages, to provide an overview of the study population and outcome variables. We then estimated the incidence of adverse perinatal outcomes (PTB, SGA, and LBW) and the trends across years. Additionally, we calculated the associated hospitalisation and/or ED presentation rates, reported as the mean or median hospitalisations and ED presentations per child-year. Finally, we described the mean or median cost per child and per admission or ED presentation from birth to age five years.

We used Generalised Additive Models (GAM) to identify the drivers of costs potentially nonlinearly associated with adverse perinatal outcomes [20, 21]

We used thin plate and cubic regression spline terms to model the smooth, non-linear relationship of cost and covariates and then checked the best fit. Finally, we found that GAM with a gamma distribution and log link function has the lowest Akaike Information Criterion (AIC) and Residual Maximum Likelihood (REML) values and a higher R-squared value, which we considered the best fit model.

Our adjustment variables were selected using a forward variable selection method. Covariates that were controlled for in GAM were selected based on published evidence including the clinical characteristics such as gestational age (weeks), birthweight (grams), Apgar score; evaluation of a newborn following birth using the criteria – Activity, Pulse, Grimace, Appearance, and Respiration[22], at one minute and five minutes, indications of congenital malformation of the baby that are present at birth (diagnosed, not diagnosed, under investigation, unknow), mode of separations of infant following birth hospitalisation (discharged to usual residence, transferred to an(other) acute hospital, died, left against advice or other), length of birth hospitalisation stay (days), length of hospital stay during the subsequent admission (days) (for hospitalisation cost), time spent at ED (hours) (for cost of ED presentation), and sociodemographic characteristics such as child sex at birth (male vs female), child (paternal) Indigenous status (Indigenous vs non-Indigenous), age of the mother at birth, remoteness of residence (remote vs urban, defined based on the Australian Statistical Geography Standard)[23] and maternal marital status (single, widowed, divorced, separated, married/de facto), and other obstetric related characteristics such as parity, number of antenatal care visits during pregnancy, and whether it is a first pregnancy.

Finally, we fitted models for hospitalisation cost (model I), cost of ED presentation (model II), and combined cost for hospitalisation and ED presentation (model III), to identify the drivers of cost as follows.

Model I

$$
\begin{aligned}
g\big(E(\text{Hospitalisation cost})\big) &= \beta_0 + \beta_1(\text{Indigenous status}) + \beta_2(\text{Remoteness}) + \beta_3(\text{Apgar score at 1 minute}) \\
&+ \beta_4(\text{Apgar score at 5 minutes}) + \beta_5(\text{ANC visit}) \\
&+ \beta_6(\text{Congenital malformation status at birth}) + \beta_7(\text{First pregnancy}) \\
&+ \beta_8(\text{Mother's marital status}) + \beta_9(mode\ of\ delivery) \\
&+ \beta_{10}(\text{Child sex assigned at birth}) \\
&+ \beta_{11}(\text{Birth weight percentiles for gestational age at birth}) + \beta_{12}(\text{Year of birth}) \\
&+ \beta_{13}(\text{Country of birth}) + \beta_{14}(Mode\ of\ separations\ follwoing\ birth\ hospitalisation) \\
&+ \beta_{15}(\text{Parity}) + \mathrm{S}(\text{Age of the mother's}) + \mathrm{S}(\text{Gestational age}) + \mathrm{S}(\text{Birthweight}) \\
&+ \mathrm{S}(\text{Length of stay following birth hospitalisation}) \\
&+ \mathrm{S}(\text{Length of stay during readmission})
\end{aligned}
$$

Where β_0 *is for intercept,* β_1 *to* β_{15} *is for parametric coefficient (for categorical and linear variables), whereas **S** is for Estimated Degree of Freedom (EDF) for smoother terms (non-linear continuous variables).*

Model II

$$
\begin{aligned}
g\big(E(\text{ED cost})\big) &= \beta_0 + \beta_1(\text{Indigenous status}) + \beta_2(\text{Remoteness}) + \beta_3(\text{Apgar score at 5 minutes}) \\
&+ \beta_4(\text{ANC visit}) + \beta_5(\text{Congenital malformation status at birth}) + \beta_6(\text{First pregnancy}) \\
&+ \beta_7(\text{Mother's marital status}) + \beta_8(\text{Child sex at birth}) \\
&+ \beta_9(\text{Mode of separation following birth hospitalisation}) + \beta_{10}(\text{Parity}) \\
&+ \beta_{11}(\text{Year of birth}) + \mathrm{S}(\text{Age of the mother's}) + \mathrm{S}(\text{Birthweight}) \\
&+ \mathrm{S}(\text{Length of stay following birth hospitalisation}) + \mathrm{S}(\text{Length of ED stay})
\end{aligned}
$$

Model III

$$
\begin{aligned}
g\big(\mathrm{E}(\text{Combined cost})\big) &= \beta_0 + \beta_1(\text{Indigenous status}) + \beta_2(\text{birthweight}) + \beta_3(\text{Remoteness}) \\
&+ \beta_4(\text{Apgar score at 1 minute}) + \beta_5(\text{Apgar score at 5 minutes}) + \beta_6(\text{ANC visit}) \\
&+ \beta_7(\text{Congenital malformation status at birth}) + \beta_8(\text{First pregnancy}) + \beta_9(\text{child age}) \\
&+ \beta_{10}(\text{Child sex at birth}) + \beta_{11}(\text{Mode of separation following birth hospitalisation}) \\
&+ \beta_{12}(\text{Parity}) + \beta_{13}(\text{Year of birth}) + \beta_{14}(\text{Age of the mother's}) \\
&+ \beta_{15}(\text{Mother'scountry of birth}) + \mathrm{S}(\text{Length of stay following birth hospitalisation}) \\
&+ \mathrm{S}(\text{Length of stay during readmission}) + \mathrm{S}(\text{Length of ED stay})
\end{aligned}
$$

Missing data handling and sensitivity analysis

We conducted a complete case analysis. We also performed subgroup and sensitivity analyses to assess the influence of outliers on our model's performance.

Our subgroup analyses were based on perinatal morbidity (PTB, SGA, and LBW). Furthermore, our sensitivity analyses evaluated the influence of extreme (outlier) values for variables such as maternal

age, gestational age, and length of hospitalisation and ED stay. For the ED time spent, we restricted our analysis to 15 minutes to 24 hours following the National Emergency Access Target in Australia[24].

Ethical approval and data access

This study received ethical approval from the Human Research Ethics Committee of the Northern Territory Health and the Menzies School of Health Research (NT HREC; Ref #: 2022-4363) and from the Curtin University Human Research Ethics Office (Ref #: HRE2024-0039). Data were accessed on 02/02/2024. All data were de-identified, and the authors had no access to any information that could identify individual participants during or after data collection.

Role of the funding source

The funder of the study had no role in the design and conduct of the study; collection, management, analysis, and interpretation of the data; or in the preparation, review, or approval of the manuscript. All authors had full access to all the data and final responsibility for the decision to submit for publication.

Results

Description of study participants

We included 58,910 singleton live births from July 1, 2000, to June 30, 2016. Of these, 31,183 births were linked to hospitalisation records, and 42,174 were linked to ED presentation records. These were included in the respective analyses of hospitalisation and ED presentation rates and its associated costs. Additionally, 27,459 births had records of both at least one hospital admission and one ED presentation; included in the combined costs of hospitalisations and ED presentations analyses.

Of these children admitted to inpatient care, 14.3% were PTB, 12.8% LBW, and 15.6% SGA, whereas among those children visited the ED before their fifth birthday, 13.5% were SGA at birth, 9.1% were born preterm, 7.9% were had LBW. The median length of hospital stays (during the subsequent admission) among these admitted was 3 (IQR: 1.4, 5.0) days (Table 1).

Table 1. Description of the study participants, NT, Australia, 2000 to 2020.

**Variables**	**Hospitalisation (n=31,183)**	**ED presentation (n=42,174)**	**Combined (hospitalisation and ED presentation) (n= 27,459)**
	n (%)	n (%)	n (%)
Gestational age			
EPTB (20^{+0} – 27^{+6} weeks)	243 (0.8)	127 (0.3)	126 (0.5)
VPTB (28^{+0} – 31^{+6} weeks)	528 (1.7)	412 (1.0)	410 (1.5)
MPTB (32^{+0} – 33^{+6} weeks)	614 (2.0)	498 (1.2)	481 (1.7)
LPTB (34^{+0} – 36^{+6} weeks)	3,060 (9.8)	2,785 (6.6)	2,496 (9.1)
Term (>=37^{+0} weeks)	26,738 (85.7)	38,352 (90.9)	23,946 (87.2)
Birthweight, mean (SD) (in grams)	3,195.2(667.1)	3,295.6(592.9)	3,218.4(638.2)
LBW			
Yes	3,999(12.8)	3,344(7.9)	3,120(11.4)

No	27,184(87.2)	38,830(92.1)	24,339(88.6)
Sex assigned at birth			
Male	17,171 (55.1)	22,454(53.2)	15,206(55.4)
Female	14,005 (44.9)	19,712(46.7)	12,248(44.6)
Others	7 (0.02)	8(0.02)	¥
Gestational age for birthweight percentiles*			
SGA	4,870 (15.6)	5,695(13.5)	4,254(15.5)
AGA	23,391 (75.0)	32,537(77.2)	20,670(75.3)
LGA	2,915 (9.4)	3,934(9.3)	2,530(9.2)
Indigenous status (for baby)			
Indigenous	17,392 (55.8)	18,762(44.5)	15,724 (57.2)
Non-Indigenous	12,592 (40.4)	20,389(48.3)	10,646 (38.8)
Unknown	1,199 (3.8)	3,023(7.2)	1,089 (4.0)
Remoteness			
Remote	14,806 (47.5)	16,213(38.5)	13,177(48.0)
Urban	16,374(52.5)	25,946(61.5)	14,280(52.0)
Mode of separations following birth hospitalisation			
Discharge to usual residence	24,797 (79.7)	36,773(87.5)	22,438(81.3)
Transferred	1,011 (3.2)	868(2.1)	816(3.0)
Died	126 (0.4)	¥	¥
Left against advice	331 (1.1)	345(0.8)	300(1.1)
Others	4,553 (14.6)	3,715(8.8)	3,598(13.1)
Unknow	302 (1.0)	323(0.8)	248(0.9)
Indigenous status of the mother			
Indigenous	17,392 (55.8)	17,193(40.8)	14,709(53.6)
Non-Indigenous	12,592 (40.4)	24,970(59.2)	12,747(46.4)
Unknown	1,199 (3.8)	11(0.03)	¥
Age of the mother, mean (SD) (in years)	26.6 (6.4)	27.2(6.3)	26.4(6.4)
Country of birth	*N=31,172*	*N=42,157*	*N=27,449*
Australia	27,620 (88.6)	36,509 (86.6)	24,502(89.3)
Others	3,468 (11.1)	5,507(13.1)	2,887(10.5)
Unknown	84 (0.3)	141(0.3)	60(0.2)
First pregnancy			
Yes	8,963(28.7)	12,165(28.8)	7,820(28.5)
No	21,938(70.4)	29,590(70.2)	19,405(70.7)
Unknown	282(0.9)	419(1.0)	234(0.8)
Parity			
Null	11,940(38.3)	16,761(39.8)	10,416(37.9)
One	8,707(27.9)	12,453(29.5)	7,731(28.2)
Two to four	9,325 (29.9)	11,641(27.6)	8,250(30.0)
Five and above	1,204(3.9)	1,308 (3.1)	1,055(3.8)

AGA: appropriate-for-gestational-age
EPTB: Extremely preterm birth
LBW: low birthweight
LGA: large-for-gestational-age
LPTB: Late preterm birth
MPTB: Moderate preterm birth
SGA: Small-for-gestational-age
VPTB: Very preterm birth
¥Values <6 are not reported as per the Human Research Ethics Committee (HREC) protocol

Trends of adverse perinatal outcomes

We observed a slight increase in the incidence of PTB from 81 in 2000 to 87 per 1,000 livebirths in 2016. A similar trend was noted for LBW, which increase marginally from 77 in 2000 to 78 per 1,000

livebirths in 2016. In contrast, the incidence of SG) at birth decreased from 152 in 2000 to 113 per 1,000 livebirths in 2016 (**Fig 2**).

Fig 2: Trends of adverse perinatal outcomes, NT, Australia, 2000 to 2016.
__A:__ Trends by adverse perinatal outcomes (SGA, PTB, and LBW), __B:__ Trends by PTB categories (EPTB, VPTB, MPTB, LPTB and Overall PTB rate), __C:__ Trends by gestational age for birthweight percentile (SGA and LGA)
EPTB: Extreme PTB (<28 weeks),
LBW: Low birthweight
LGA: Large-for-gestational-age
LPTB: Late preterm birth (35^{+0} to 36^{+6} weeks)
MPTB: Moderate preterm birth (33^{+0} to 34^{+6} weeks)
PTB: Preterm births
SGA: Small-for-gestational-age
VPTB: Very preterm birth (28^{+0} to 32^{+6} weeks)

Hospitalisation and ED presentation

We found that the overall mean number of hospitalisations per child was 3.1 (SD: 4.1) over the first five years. The mean number of hospitalisations per preterm child was 3.1 (SD: 3.5), per LBW child 3.2 (SD: 3.5), SGA was 2.8 (SD: 3.0), and per preterm child with LBW and SGA was 3.8 (SD: 3.4), while per term child with no LBW and SGA was 2.2 (SD: 2.3). Between 2000 and 2020, the mean hospitalisation increased for PTB (1.3 (SD:0.7) to 6.9 (SD: 6.0)), LBW births (1.3, SD: 0.6 to 7.2, SD: 5.2), and SGA births (1.2, SD: 0.6 to 8.1, SD: 15.1) in the first five years, with an exponential increase among children with SGA followed by LBW and PTB (**Fig 3a**). We found increasing hospitalisation rate over time, particularly 2017 onwards in all adverse perinatal outcomes. We found the overall median ED presentation per child was 3 (IQR: 1 to 6) over the first five years. Our findings also suggested that there is an increasing ED presentation across all adverse perinatal outcomes with a large change after the 2016. Children born preterm and with LBW presented to the ED more frequently than these children born SGA (**Fig 3b**). On the contrary, we observed a decreasing trend in hospital stays with each subsequent hospitalisation, while the length of stay after birth hospitalisation and ED presentations remained consistent across years for all adverse perinatal outcomes (Fig S1).

Fig 3. Trends of hospitalisation and ED presentation by adverse perinatal outcomes, NT, Australia, 2000 to 2020.

Cost of hospitalisation and ED presentation

We found that the overall median hospitalisation cost per preterm child with LBW and SGA was AUD 23,849 (IQR: 11,858–44,475), cost per child with at least one adverse perinatal outcome was AUD 12,373 (5,998–26,347), while the cost per term child with no SGA and LGA was AUD 8,668 (IQR: 4,365–17,855) in the first five years. Between 2000 and 2020, the median hospitalisation costs increased for SGA births (AUD 3,807, IQR: 3,527–6,175 to AUD 5,245, IQR: 3,016–9,931) and LBW births

(AUD 5,234, IQR: 3,807–9,955 to AUD 5,843, IQR: 2,398–10959), while cost for PTB was decreased, from AUD 6,581 (IQR: 3,807–11,368) to AUD 6,219 (IQR: 4,365–10,959). We found that the median hospitalisation cost per child born preterm within the first five years of life was AUD 19,362 (IQR: 9,056–35,339), while the cost per child born term was AUD 8,887 (IQR: 4,579–18,713) (Table S1). The median hospitalisation cost per term child with AGA and normal birthweight was AUD 8,668 (IQR: 4,365 –17,855), while the median cost per child with at least one morbidity was AUD 12,373 (IQR: 5,998 – 26,347). The mean cost for a child with all adverse perinatal outcomes (PTB, LBW, and SGA) was twice as high as the total mean cost of all births, while the mean cost for a child with at least one adverse perinatal outcome was 1.2 times higher (Table S2). Based on the classification of preterm, the median hospitalisation cost per child per year for EPTB, VPTB, MPTB and LPTB were AUD 8,687 (IQR: 5,237–27,764), AUD 9,871 (IQR: 5,700–19,140), AUD 8,088 (IQR: 4,677–13,641), and AUD 6,500 (IQR: 4,311–9,620), respectively (Table S3-S5). The majority of the hospitalisation costs were incurred in the first year, with costs decreasing in subsequent years (**Fig 4**). However, we observed an increasing trend in hospitalisation cost over time till 2016 and then onwards a slight decreasing (**Fig 5**, Fig S2 and S3).

Fig 4. Hospitalisation cost by child age and adverse perinatal outcomes, NT, Australia, 2000 to 2020.
APO: Adverse Perinatal outcomes (PTB, LBW, and SGA)
LBW: Low birthweight
PTB: Preterm births
SGA: Small-for-gestational-age

Fig 5. Trends of hospitalisation cost by adverse perinatal outcomes, NT, Australia, 2000 to 2020.

We found the median cost of ED presentation per preterm child with LBW and SGA in the first five years of age was AUD 3,108 (IQR: 1,609 –7,520), while the cost per a term child without these outcomes was AUD 2,058 (IQR: 1,032 – 4,057). Between 2000 and 2020, the median cost of ED presentation for preterm child with SGA and LBW was increased from AUD 743 (IQR: 596-890) to AUD 815 (IQR: 599-990), compared with a term child with no SGA and LBW decreased from AUD 890 (IRQ: 823-890) to AUD 690 (IQR: 561-885) (Table S6). The mean cost of ED presentation increased for SGA births (AUD 803 ± 147 to AUD 853 ± 355), while cost for LBW births (AUD 846 ± 107 to AUD 818 ± 322) and PTB (AUD 845 ± 108 to AUD 793 ± 295) was decreased (Table S7). We observed an increase in the mean cost of ED presentations per EPTB child per year over time (Table S8). The mean cost of ED presentation has been decreasing across child age in all adverse perinatal outcomes (**Fig 6**).

Fig 6. Cost of ED presentations by child age and adverse perinatal outcomes, NT, Australia, 2000 to 2020.
APO: Adverse Perinatal outcomes (PTB, LBW, and SGA, LBW: Low birthweight, PTB: Preterm births, SGA: Small-for-gestational-age

Overall, the median cost (combined cost of hospitalisation and ED presentation) per a preterm child with SGA and LBW within the first five years of age was AUD 31,210 (IQR: 17,793); while the median cost per a term child without LBW and SGA was AUD 9,007 (IQR: 4,689–18,485). The cost has been decreasing across child age for all adverse outcomes (**Fig 7**).

Fig 7. Cost of hospitalisation and ED presentation by child age and adverse perinatal outcomes, NT, Australia, 2000 to 2020.
APO: Adverse Perinatal outcomes (PTB, LBW, and SGA), LBW: Low birthweight, PTB: Preterm births, SGA: Small-for-gestational-age

Drivers of cost of hospitalisation and ED presentation

We found that, after adjustment, hospitalisation cost was 34.8% (95% CI: 32.5 to 37.2) higher for children from Indigenous families compared to those children from non-Indigenous families. Hospitalisation costs were 8.2% (95% CI: 5.1 to 11.0) higher for children from rural areas compared to those from urban areas. A one-point increase in Apgar scores at 1 minute was associated with a 1.3% (95% CI: 0.3 to 2.3) reduction in hospitalisation cost. Children diagnosed and under investigation for congenital abnormalities incurred 44.7% (95% CI: 28.8 to 62.8) and 44.9% (95% CI: 32.8 to 58.2) higher cost of hospitalisation than other children. The birth not being the first for the mother was associated with higher hospitalisation costs by 4.1% (95% CI: 0.5 to 7.8) and a one child increase in parity was associated with a 2.5% (95% CI: 1.3 to 3.7) increase in hospitalisation cost. Emergency caesarean delivery was associated with a 4.3% (95% CI: 0.6 to 8.1) higher hospitalisation cost compared to spontaneous vaginal delivery. We observed a trend of increasing hospitalisation costs associated with adverse perinatal outcomes over time, with costs rising by up to 48.3% (95% CI: 33.1 to 65.4) in 2016 compared to the year 2000 (Table 2, Table S9).

As shown in **Figs 8A-E**, we observed strong evidence for non-linear associations between hospitalisation cost and length of hospital stay, both following birth hospitalisation (EDF = 4.33) and during subsequent admissions (EDF = 8.89, as well as maternal age (EDF = 2.55) (Tables S9-10). Specifically, the association between length of hospital stay during subsequent admissions and cost was non-linear, with a peak was noted around two weeks days, followed by a slight decrease and another increase after approximately one month of age.

Fig 8. Non-linear relationship between hospitalisation cost and covariates, NT, Australia, 2000 to 2020.

The association between birthweight and hospitalisation cost varied by gestational age. We observed that within certain ranges of birthweight (less than 3000 grams) and gestational age (less than 35 weeks), a decrease in birthweight was associated with a substantial increase in cost, particularly at lower gestational ages (Fig S4). Correspondingly, the variability in the non-linear relationship between birthweight and gestational age increased significantly at the lower extremes of both birthweight and gestational age.

Table 2: Drivers of hospitalisation cost from birth to age five years, NT, Australia, 2000 to 2020.

Variables	% Change	Lower 95% CI	Upper 95% CI
Indigenous status of mother			
Indigenous	Ref.		
Non-Indigenous	-35	-37.2	-32.3***
Remoteness of residence			
Rural	Ref.		
Urban	-8.2	-11.0	-5.1***
Apgar score at 1 minute	-1.3	-2.3	-0.3*
Apgar score at 5 minutes	-1.2	-3.0	0.6
Frequency of Antenatal care visits	-0.1	-0.3	0.3
Congenital malformation at birth			
Not diagnosed	Ref.		
Diagnosed	44.7	28.8	62.8***
Under investigation	44.9	32.8	58.2***
Unknown	6.7	-0.1	13.9
First pregnancy			
Yes	Ref.		
No	4.1	0.5	7.8**
Mother's marital status			
Single	Ref.		
Married	12.2	8.9	15.4***
Others#	25.2	18.4	32.5***
Outcome following birth hospitalisation			
Discharge to usual residence	Ref.		
Transferred to acute care facility	54.3	42.1	67.6***
Left against advice	16.0	2.7	30.9*
Others	9.3	5.3	13.5***
Unknown	24.0	5.7	45.3**
Sex at birth			
Male	Ref.		
Female	-8.0	-10.3	-5.6***
Parity	2.5	1.3	3.7***
Mode of delivery			
SVD	Ref.		
Breech	8.2	-15.0	37.7
Forceps	1.1	-7.0	9.8
Ventouse	-2.4	-8.0	3.6
CS elective	-1.9	-6.2	2.4
CS emergency	4.3	0.6	8.1*
Birthweight for gestational age percentiles			
SGA	3.8	-1.5	9.2

AGA	Ref.		
LGA	1.0	-5.4	7.6
Year of birth			
2000	Ref.		
2001	5.0	-4.0	15.0
2002	11.5	1.7	22.3*
2003	16.3	5.6	28.1**
2004	27.1	15.4	40.0***
2005	26.5	15.0	39.2***
2006	28.8	17.1	41.7***
2007	33.0	20.8	46.3***
2008	27.5	16.0	40.2***
2009	38.8	26.2	52.6***
2010	33.1	21.1	46.3***
2011	35.2	23.0	48.7***
2012	39.6	27.0	53.5***
2013	41.9	29.1	56.0***
2014	42.2	29.5	56.3***
2015	49.6	36.2	64.5***
2016	48.3	33.1	65.4***
Mother's country of birth			
Australia	Ref.		
Others	-2.0	-5.3	3.4

*Significant codes: *** < 0.001, ** < 0.01, *< 0.05*
AGA: Appropriate-for-gestational-age
CS: Caesarean Section
LGA: Large-for-gestational-age
SGA: small-for-gestational-age
SVD: Spontaneous vaginal delivery

We found that cost of ED presentation was 9.3% (95% CI: 8.3 to 10.3) higher for children from Indigenous families compared to those children from non-Indigenous families. The cost was 5.1% (95% CI: 4.2 to 6.0) lower for children from urban areas compared to those from rural areas. A one-point increase in Apgar scores at 5 minutes was associated with a 1.3% (95% CI: 0.7 to 1.9) reduction in ED presentation costs. Children diagnosed and under investigation for congenital abnormalities incurred 4.8% (95% CI: 0.6 to 9.2) and 5.7% (95% CI: 2.6 to 8.9) cost of ED presentation than children who were not diagnosed congenital anomalies, respectively. We observed a trend of increasing cost of ED presentation associated with adverse perinatal outcomes over time, with costs rising by up to 18.0% (95% CI:13.8 to 22.1) in 2016 compared to the year 2000 (Table 3, Table S11).

As shown in Figs **9A-E**, we observed significant non-linear associations between cost of ED presentation and length of hospital stay, both following birth hospitalisation (EDF= 2.62) and ED presentation (EDF= 4.31), birthweight (EDF= 4.93) as well as maternal age (EDF= 3.82) (Tables S11-12). The association between birthweight and cost of ED presentation varied by gestational age. We observed that in the lower ranges of birthweight and gestational age, a substantial increase in cost of ED presentation (Fig S5). In the combined cost drivers analysis, we identified a range of covariates that affect the cost (Table S13).

Fig 9: Non-linear relationship of cost of ED presentation and covariates, NT, Australia, 2000 to 2020.

Table 3: Drivers of cost of ED presentation from birth to age five years, NT, Australia, 2000 to 2020.

Variables	% Change	Lower 95% CI	Upper 95% CI
Indigenous status of baby			
Indigenous	Ref.		
Non-Indigenous	-9.3	-10.3	-8.3***
Remoteness of residence			
Rural	Ref.		
Urban	-5.1	-6.0	-4.2***
Apgar score at 1 minute	0.3	-0.1	0.6
Apgar score at 5 minutes	-1.3	-1.9	-0.7***
Antenatal care visits	-0.1	-0.2	0.0**
Congenital malformation at birth			
Not diagnosed	Ref.		
Diagnosed	4.8	0.6	9.2*
Under investigation	5.7	2.6	8.9***
Unknown	3.9	1.7	6.2***
First pregnancy			
Yes	Ref.		
No	2.3	1.2	3.3***
Mother's marital status			
Single	Ref.		
Married	1.4	0.4	2.3**
Others	1.7	0.1	3.3*
Unknown	2.8	-2.0	7.9
Outcome of admission following birth			
Discharge to usual residence	Ref.		
Transferred to an(other) acute care facility	-0.1	-3.0	2.9
Left against advice	9.8	5.4	14.4***
Other	6.1	4.6	7.5***
Unknown	5.4	-0.1	11.2
Sex at birth			
Male	Ref.		
Female	-1.7	-2.5	-0.9***
Parity	1.5	1.1	1.9***
Mode of delivery			
SVD	Ref.		
Forceps	-2.0	-4.4	0.4
Ventouse	0.1	-1.7	1.8
CS elective	-0.8	-2.1	0.4
CS emergency	-1.2	-2.3	0.0
Birthweight for gestational age percentiles			
SGA	-0.9	-2.7	0.9
AGA	Ref.		
LGA	0.4	-1.7	2.6
Year of birth			
2000	Ref.		
2001	-4.9	-7.8	-2.0**
2002	-6.9	-9.8	-3.9***
2003	-6.2	-9.1	-3.1***
2004	-3.9	-6.9	-0.7*
2005	-3.0	-6.1	0.1
2006	-3.1	-6.1	0.1

2007	-3.5	-6.6	-0.4*
2008	-2.5	-5.5	0.6
2009	-1.6	-4.6	1.6
2010	0.7	-2.4	3.9
2011	0.6	-2.4	3.8
2012	3.0	0.0	6.4
2013	10.0	6.6	13.5***
2014	13.6	10.1	17.2***
2015	16.0	12.4	19.6***
2016	18.0	13.8	22.1***

*Significant codes: *** < 0.001, ** < 0.01, *< 0.05*
AGA: Appropriate-for-gestational-age
CS: Caesarean Section
ED: Emergency Department
LGA: Large-for-gestational-age
SGA: small-for-gestational-age
SVD: Spontaneous vaginal delivery

Discussion

Our study is the first to comprehensively assess long-term trends in adverse perinatal outcomes, such as PTB, SGA, and LBW, alongside their associated hospitalisation, ED presentation rates and healthcare costs from birth to age five years in NT, Australia. We identified adverse perinatal outcome rates (per 1,000 livebirths) of 87 for PTB, 128 for SGA infants, and 77 for LBW infants, contributing to rising healthcare costs over the last two decades. Although healthcare costs per child are comparable to those in other Australian States and Territories, the relative burden on the NT healthcare system is higher. The total proportional cost of morbidity (a child with at least one adverse perinatal outcome) is 45% greater when compared to children without morbidity (term children without LBW and SGA), highlighting the economic challenges of these adverse perinatal outcomes to the health system in this region. By the age of five years, the median healthcare cost for a preterm child with SGA and LBW was AUD 31,210, which included AUD 23,848 for hospitalisations and AUD 3,108 for ED presentations, compared with a term child without LBW and SGA was AUD 9,007 including hospitalisation cost of AUD 8,668 and ED cost of AUD 2,058. Between 2000 and 2020, median hospitalisation costs rose for SGA (AUD 3,807 to AUD 5,245) and LBW births (AUD 5,234 to AUD 5,843) but declined for PTB births (AUD 6,581 to AUD 6,219). Median ED costs increased for preterm children with SGA and LBW (AUD 743 to AUD 815) but decreased for term children without these outcomes (AUD 890 to AUD 690). These results highlight the dual health and financial burden associated with adverse perinatal outcomes during early childhood. Addressing these challenges through preventative strategies and early interventions is essential to reduce healthcare costs and improve long-term health outcomes [25-28].

Our findings show an increasing trend in PTB and LBW, while a slight decrease in SGA was observed over time. This is consistent with previous studies, which reported a slight increasing trends in PTB, particularly among Indigenous populations[5], as well as a study by Yeung et al.[29] in Canada. Similarly, we observed an increase in hospitalisations and ED presentations over the past decades,

particularly from 2016 onwards. Our findings are supported by other studies from other Australian states[7] and the US[30], which also reported higher hospitalisation rates among children with adverse perinatal outcomes such as PTB. The higher rates of hospitalisation and ED presentation observed in our study may partly be attributed to the mumps outbreak in the regional Australia, particularly in the Northern and Western Australia, from 2015 to 2017[31, 32], which increased hospital visits. In contrast, our findings show a decrease in the length of stay per readmission over the past decades for all adverse perinatal outcomes, aligning with a study in Canada[33]. However, a contrasting study from the same country reported an increase in the length of stay for extreme PTB (<24 weeks)[29]. Other research found no association between length of hospital stay and the risk of readmission[34, 35]. We observed a consistent pattern in the length of hospital stay following birth hospitalisation and ED presentations. The median length of stays following birth hospitalisation was longer than subsequent admissions, particularly for children born LBW and PTB, compared to term children and those with appropriate birthweight. Previous studies have similarly shown that children born with adverse birth outcomes tend have longer hospital stays before discharge[7]. Conversely, the length of birth hospitalisation, along with subsequent admissions and ED presentations, required greater levels of intervention and were more intensive in the period immediately surrounding the time of birth. Our findings also suggest that longer hospital stays increase hospitalisation costs.

Certain clinical characteristics of the child are associated with increased healthcare costs. We observed a non-linear relationship between costs and factors such as gestational age, birthweight, length of hospital stays (including both birth hospitalisation and subsequent admission or ED stays), and child age. Birth at extreme gestational ages and birthweight were linked with increased financial cost for the healthcare system. Specifically, the impact of LBW on hospitalisation cost is more pronounced in preterm infants, highlighting the importance of targeted interventions for preventing PTB and LBW such as corticosteroids and progesterone administration for high-risk pregnancies, as well as nutritional support during pregnancy[36-39], to prevent PTB and LBW. Healthcare costs initially decrease, then peak around two weeks of age before gradually decreasing again as the child aged. The observed non-linear relationship between cost and child age suggests that while post-birth hospitalisation discharge costs may decrease, infants remain vulnerable to infections, complications related to congenital conditions, and issues with feeding or growth, all of which contribute to ongoing healthcare needs. Previous studies support our findings that costs are notably higher for neonates born extremely and very preterm compared to those born late preterm, as they require more support during their initial hospitalisation[40]. Furthermore, infants born extremely (20 to 27^{+6} weeks) and very preterm (28^{+0} to 32^{+6} weeks) are more likely to develop comorbidities than those children born at full term, both in the short-term and later in life[41-43]. Infants born extremely preterm require frequent follow-up visits as they age, leading to continued healthcare costs over time[11, 44]. We found that more than a third of the total cost related to adverse perinatal outcome has been incurred in the first few months following

births, and then as their age increases, the direct and indirect cost to the health system reduces. This finding is supported by a study by Jacob et al. (2017),[45] which examined the cost effects of PTB, and showed that the cost decreases as the child age increases. Other studies have also identified that children's clinical characteristics affect the cost of hospitalisations among children with adverse outcomes[46, 47]. This might be explained by the fact that infants born preterm and with LBW often require longer hospital stays after birth to gain the necessary weight and maturity before discharge, which places an additional burden on healthcare resources[48].

Vulnerable populations, particularly children of Indigenous parents and those residing in remote areas, incur higher healthcare costs compared to the average, which may place a disproportionate burden on the healthcare system. Our findings are consistent with another study in Australia, which highlight the higher healthcare utilisation and costs among vulnerable children during their early childhood years[49].

The strengths of our study include the use of a two-decade, population-based data, which allows for estimation of long-term trends in adverse perinatal outcomes and their associated hospitalisation and ED presentations and financial burden on the healthcare system. We included a broader range of adverse outcomes, encompassing major perinatal morbidity, and examined costs from birth up to age five, as this period captures most childhood morbidity following adverse outcomes[50, 51] and reflects the long-term burden across decades.

Our study has some notable limitations. We deflated the costs back to 2000 using the 2012 CPI as the base year. However, this approach may not account for short-term fluctuations in healthcare costs resulting from economic disruptions or policy changes, nor does it fully capture advancements in care efficiency or the increasing use of costly medical innovations over time. We estimated the child's age using the first day of the month as the date of birth, because the NT Perinatal Trends dataset did not include exact birthdates. This approach may result in hospitalisations being misclassified into older age groups (e.g., assigning a hospitalisation to "28 days of age" when it occurred during the early neonatal period). Similarly, costs associated with neonatal care could be attributed to later age intervals, which might inflate the healthcare burden for older age groups. Our cost estimation included only hospitalisation and ED presentations, excluding costs from GP or physician visits, which may lead to an underestimation of healthcare costs associated with adverse perinatal outcomes. We assumed that all births occurring in the state remained there and included them in our cost estimation. However, interstate and international migration may affect our estimation of hospitalisation, ED presentations and costs, probably leading to an underestimation[52]. The final limitation of our study, due to the unavailability of socioeconomic variable before 2013 in the Perinatal Trends dataset, is that we did not adjust for it in our model of cost drivers identification.

The stagnation of improvements in adverse perinatal outcomes such as PTB and LBW children in the NT over the past decades has placed a health burden and increased costs for the healthcare system through frequent hospital visits. The financial burden to the healthcare system is largely driven by extended hospital stays, vulnerability such as remote areas and Indigenous status, extreme of gestational age and birthweight, and also diagnosed and suspected congenital malformation. Thus, enhancing perinatal health interventions to shorten the length of birth hospitalisation and subsequent admission and ED presentation is worthwhile, as our findings suggest that decreasing length of stay could lead to significant healthcare resource savings. Furthermore, vulnerable populations, particularly children born to Indigenous parents and those living in remote areas, incur higher healthcare costs than the average. These findings highlight the need for further research to investigate the association between length of stay and the risk of readmission among children with adverse perinatal outcomes. Additionally, quantifying the disparity of the burden across different population groups is essential to enable more targeted interventions aimed at reducing the associated dual health and economic impacts.

Data availability statement

The dataset is the property of the Northern Territory Department of Health, Australia, and is not public available. Access to the datasets should be upon request and approval of ethics committee and data custodians and requests can be directed to SA-NT data link (https://www.santdatalink.org.au/).

Declaration of interests

The authors have declared that no competing interests exist.

Funding

This research was supported by the Australian National Health and Medical Research Council (NHMRC) Investigator Grant (#1195716) to GAT, and NHMRC Project (#1099655) and investigator grant (#1173991) GP. TGH is supported by Research Training Programme linked to the NHMRC.

Acknowledgements

The authors would like to acknowledge the SA NT DataLink personnel for their technical and administrative assistance in linking the datasets and thank all data custodians for their support in data retrieval, preparation, and release. The authors also acknowledge funding from the Australian National Health and Medical Research Council (NHMRC) through project and Investigator Grants. TGH is supported by Research Training Programme linked to the NHMRC and acknowledges Curtin University for providing the support.

References

1. Liang X, Lyu Y, Li J, Li Y, Chi C. Global, regional, and national burden of preterm birth, 1990-2021: a systematic analysis from the global burden of disease study 2021. eClinicalMedicine. 2024;76. doi: 10.1016/j.eclinm.2024.102840.

2. Ohuma EO, Moller A-B, Bradley E, Chakwera S, Hussain-Alkhateeb L, Lewin A, et al. National, regional, and global estimates of preterm birth in 2020, with trends from 2010: a systematic analysis. The Lancet. 2023;402(10409):1261-71.
3. Okwaraji YB, Krasevec J, Bradley E, Conkle J, Stevens GA, Gatica-Domínguez G, et al. National, regional, and global estimates of low birthweight in 2020, with trends from 2000: a systematic analysis. The Lancet. 2024;403(10431):1071-80.
4. Hug L, You D, Blencowe H, Mishra A, Wang Z, Fix MJ, et al. Global, regional, and national estimates and trends in stillbirths from 2000 to 2019: a systematic assessment. The Lancet. 2021;398(10302):772-85.
5. Brown K, Cotaru C, Binks M. A retrospective, longitudinal cohort study of trends and risk factors for preterm birth in the Northern Territory, Australia. BMC Pregnancy and Childbirth. 2024;24(1):33. doi: 10.1186/s12884-023-06164-6.
6. Perin J, Mulick A, Yeung D, Villavicencio F, Lopez G, Strong KL, et al. Global, regional, and national causes of under-5 mortality in 2000–19: an updated systematic analysis with implications for the Sustainable Development Goals. Lancet Child Adolesc Health. 2022;6(2):106-15. doi: 10.1016/S2352-4642(21)00311-4.
7. Stephens A, S r, Lain SJ, Roberts CL, Bowen JR, Nassar N. Survival, hospitalization, and acute-care costs of very and moderate preterm infants in the first 6 years of life: a population-based study. The Journal of pediatrics. 2016;169:61-8. e3. doi: doi:. PubMed PMID: rayyan-998549172.
8. Health AIo, Welfare. Australia's mothers and babies. Canberra: AIHW, 2024.
9. Oster ME, Dawson AL, Batenhorst CM, Strickl, MJ, Kleinbaum DG, et al. Relationship between resource utilization and length of stay following tetralogy of fallot repair. Congenit. 2013;8(6):535-40. doi: doi:https://dx.doi.org/10.1111/chd.12023. PubMed PMID: rayyan-985252311.
10. Glinianaia SV, Morris JK, Best KE, Santoro M, Coi A, Armaroli A, et al. Long-term survival of children born with congenital anomalies: A systematic review and meta-analysis of population-based studies. PLoS medicine. 2020;17(9):e1003356.
11. Callander EJ, Atwell K. The healthcare needs of preterm and extremely premature babies in Australia—assessing the long-term health service use and costs with a data linkage cohort study. Eur J Pediatr. 2021;180:2229-36.
12. Demetriou EA, Boulton KA, Thapa R, Sun C, Gilroy J, Bowden MR, et al. Burden of paediatric hospitalisations to the health care system, child and family: a systematic review of Australian studies (1990–2022). The Lancet Regional Health - Western Pacific. 2023;40:100878. doi: https://doi.org/10.1016/j.lanwpc.2023.100878.
13. Australian Bureau of Statistics 2021 PC, ABS, viewed 11 December 2024, <https://www.abs.gov.au/statistics/people/population/population-census/latest-release>.
14. Karácsonyi D, Taylor A. Reconceptualising sparsely populated remote edges through the concept of region and space. Journal of Rural Studies. 2022;92:395-403. doi: https://doi.org/10.1016/j.jrurstud.2022.05.001.
15. Health AIo, Welfare. Australian refined diagnosis-related groups (AR-DRG) data cubes. Canberra: AIHW, 2024.
16. Vogel JP, Chawanpaiboon S, Watananirun K, Lumbiganon P, Petzold M, Moller A-B, et al. Global, regional and national levels and trends of preterm birth rates for 1990 to 2014: protocol for development of World Health Organization estimates. Reproductive Health. 2016;13(1):76. doi: 10.1186/s12978-016-0193-1.
17. Dobbins TA, Sullivan EA, Roberts CL, Simpson JM. Australian national birthweight percentiles by sex and gestational age, 1998–2007. Medical Journal of Australia. 2012;197(5):291-4.
18. Rice DP. Cost-of-illness studies: fact or fiction? Lancet. 1994;344(8936):1519-20. Epub 1994/12/03. doi: 10.1016/s0140-6736(94)90342-5. PubMed PMID: 7983947.
19. Australian Bureau of Statistics. Consumer Price Index Australia [Internet]. Canberra: ABS; Sep-quarter-2024 [cited 2024 December 11]. Available from: https://www.abs.gov.au/statistics/economy/price-indexes-and-inflation/consumer-price-index-australia/l.
20. Hastie TJ. Generalized additive models. Statistical models in S: Routledge; 2017. p. 249-307.
21. Wood SN. Generalized additive models: an introduction with R: chapman and hall/CRC; 2017.

22. Fetus AAoPCo, Newborn, Obstetricians ACo, Practice GCoO, Watterberg KL, Aucott S, et al. The apgar score. Pediatrics. 2015;136(4):819-22.

23. Australian Bureau of Statistics. Remoteness Areas [Internet]. Canberra: ABS; Jul2021-Jun2026 [cited 2024 December 11] Afhwagassa-s.

24. Sullivan C, Staib A, Griffin B, Bell Jr A, Scott I. The four hour rule: the National Emergency Access Target in Australia, time to review, systematic literature review appendix. Aust Health Rev. 2016;40(3):319.

25. Bhutta ZA, Das JK, Bahl R, Lawn JE, Salam RA, Paul VK, et al. Can available interventions end preventable deaths in mothers, newborn babies, and stillbirths, and at what cost? The Lancet. 2014;384(9940):347-70. doi: 10.1016/S0140-6736(14)60792-3.

26. Darmstadt GL, Bhutta ZA, Cousens S, Adam T, Walker N, de Bernis L. Evidence-based, cost-effective interventions: how many newborn babies can we save? The Lancet. 2005;365(9463):977-88. doi: 10.1016/S0140-6736(05)71088-6.

27. Chang HH, Larson J, Blencowe H, Spong CY, Howson CP, Cairns-Smith S, et al. Preventing preterm births: analysis of trends and potential reductions with interventions in 39 countries with very high human development index. The Lancet. 2013;381(9862):223-34. doi: 10.1016/S0140-6736(12)61856-X.

28. Sebastian E, Bykersma C, Eggleston A, Eddy KE, Chim ST, Zahroh RI, et al. Cost-effectiveness of antenatal corticosteroids and tocolytic agents in the management of preterm birth: A systematic review. eClinicalMedicine. 2022;49. doi: 10.1016/j.eclinm.2022.101496.

29. Yeung T, Rios JD, Beltempo M, Khurshid F, Toye J, Ojah C, et al. The Trend in Costs of Tertiary-Level Neonatal Intensive Care for Neonates Born Preterm at 220/7-286/7 Weeks of Gestation from 2010 to 2019 in Canada. Journal of Pediatrics. 2022;245:72-80.e6. doi: doi:https://dx.doi.org/10.1016/j.jpeds.2022.02.055. PubMed PMID: rayyan-985253006.

30. Amsalu R, Oltman SP, Baer RJ, Medvedev MM, Rogers EE, Jelliffe-Pawlowski L. Incidence, risk factors, and reasons for 30-day hospital readmission among healthy late preterm infants. Hosp. 2022;12(7):639-49.

31. Westphal DW, Eastwood A, Levy A, Davies J, Huppatz C, Gilles M, et al. A protracted mumps outbreak in Western Australia despite high vaccine coverage: a population-based surveillance study. Lancet Infect Dis. 2019;19(2):177-84. doi: 10.1016/S1473-3099(18)30498-5.

32. Walker J, Adegbija O, Smoll N, Khan A, Whicker J, Carroll H, et al. Epidemiology of mumps outbreaks and the impact of an additional dose of MMR vaccine for outbreak control in regional Queensland, Australia, 2017-2018. 2021.

33. Liu S, Wen SW, McMillan D, Trouton K, Fowler D, McCourt C. Increased Neonatal Readmission Rate Associated with Decreased Length of Hospital Stay at Birth in Canada. Canadian Journal of Public Health. 2000;91(1):46-50. doi: 10.1007/BF03404253.

34. Harron K, Gilbert R, Cromwell D, Oddie S, van der Meulen J. Newborn length of stay and risk of readmission. Paediatric and perinatal epidemiology. 2017;31(3):221-32.

35. Goyal N, Zubizarreta JR, Small DS, Lorch SA. Length of Stay and Readmission Among Late Preterm Infants: An Instrumental Variable Approach. Hosp. 2013;3(1):7-15. doi: 10.1542/hpeds.2012-0027.

36. Meis PJ, Klebanoff M, Thom E, Dombrowski MP, Sibai B, Moawad AH, et al. Prevention of recurrent preterm delivery by 17 alpha-hydroxyprogesterone caproate. New England Journal of Medicine. 2003;348(24):2379-85.

37. Crump C, Sundquist J, Winkleby MA, Sundquist K. Preterm birth and risk of chronic kidney disease from childhood into mid-adulthood: national cohort study. BMJ. 2019;365:l1346. doi: 10.1136/bmj.l1346.

38. Care A, Nevitt SJ, Medley N, Donegan S, Good L, Hampson L, et al. Interventions to prevent spontaneous preterm birth in women with singleton pregnancy who are at high risk: systematic review and network meta-analysis. Bmj. 2022;376.

39. Fonseca Eduardo B, Celik E, Parra M, Singh M, Nicolaides Kypros H. Progesterone and the Risk of Preterm Birth among Women with a Short Cervix. New England Journal of Medicine. 357(5):462-9. doi: 10.1056/NEJMoa067815.

40. Rolnitsky A, Unger SL, Urbach DR, Bell CM. Cost of neonatal intensive care for extremely preterm infants in Canada. Translational Pediatrics. 2021;10(6):1630-6. doi: doi:https://dx.doi.org/10.21037/tp-21-36. PubMed PMID: rayyan-985248302.
41. Anderson PJ, de Miranda DM, Albuquerque MR, Indredavik MS, Evensen KAI, Van Lieshout R, et al. Psychiatric disorders in individuals born very preterm / very low-birth weight: An individual participant data (IPD) meta-analysis. eClinicalMedicine. 2021;42. doi: 10.1016/j.eclinm.2021.101216.
42. Bolk J, Farooqi A, Hafström M, Åden U, Serenius F. Developmental Coordination Disorder and Its Association With Developmental Comorbidities at 6.5 Years in Apparently Healthy Children Born Extremely Preterm. JAMA Pediatrics. 2018;172(8):765-74. doi: 10.1001/jamapediatrics.2018.1394.
43. Woodward LJ, Moor S, Hood KM, Champion PR, Foster-Cohen S, Inder TE, et al. Very preterm children show impairments across multiple neurodevelopmental domains by age 4 years. Archives of Disease in Childhood-Fetal and Neonatal Edition. 2009;94(5):339-44.
44. Korvenranta E, Lehtonen L, Rautava L, Häkkinen U, Andersson S, Gissler M, et al. Impact of very preterm birth on health care costs at five years of age. Pediatrics. 2010;125(5):e1109-e14. doi: doi:. PubMed PMID: rayyan-998549144.
45. Jacob J, Lehne M, Mischker A, Klinger N, Zickermann C, Walker J. Cost effects of preterm birth: a comparison of health care costs associated with early preterm, late preterm, and full-term birth in the first 3 years after birth. The European Journal of Health Economics. 2017;18(8):1041-6. doi: 10.1007/s10198-016-0850-x.
46. Chou FS. Assessment of social factors influencing hospitalization cost of US preterm newborns, 2016. J Matern Fetal Neonatal Med. 2022;35(10):1978-86. doi: doi:10.1080/14767058.2020.1776252. PubMed PMID: rayyan-1065338166.
47. Yu S, Lui K, Fiebig DG, Travadi J, Homer CSE, Sinclair L, et al. Preterm birth and total health care use and costs in the first 5 years of life: a population-based study. Journal of Pediatrics. 2023;258(17). doi: http://dx.doi.org/10.1016/j.jpeds.2023.01.006. PubMed PMID: 20231603943.
48. Schneuer FJ, Demetriou E, Bond D, Lain SJ, Guastella AJ, Nassar N. Child characteristics and health conditions associated with paediatric hospitalisations and length of stay: a population-based study. The Lancet Regional Health – Western Pacific. 2023;32. doi: 10.1016/j.lanwpc.2023.100706.
49. Bull C, Howie P, Callander EJ. Inequities in vulnerable children's access to health services in Australia. BMJ Global Health. 2022;7(3):e007961.
50. Lawn JE, Blencowe H, Oza S, You D, Lee AC, Waiswa P, et al. Every Newborn: progress, priorities, and potential beyond survival. The lancet. 2014;384(9938):189-205.
51. Liu L, Oza S, Hogan D, Chu Y, Perin J, Zhu J, et al. Global, regional, and national causes of under-5 mortality in 2000–15: an updated systematic analysis with implications for the Sustainable Development Goals. The Lancet. 2016;388(10063):3027-35.
52. Thurmer J, Taylor A. Internal Return Migration and the Northern Territory: New Migration Analysis for Understanding Population Prospects for Sparsely Populated Areas. Population Research and Policy Review. 2021;40(4):795-817. doi: 10.1007/s11113-020-09616-5.

Fig 1. Cohort selection flow diagram, NT, Australia, 2000 to 2016. Click here to access/download;Figure;Fig 1. Cohort selection flow diagram, NT, Australia.tif

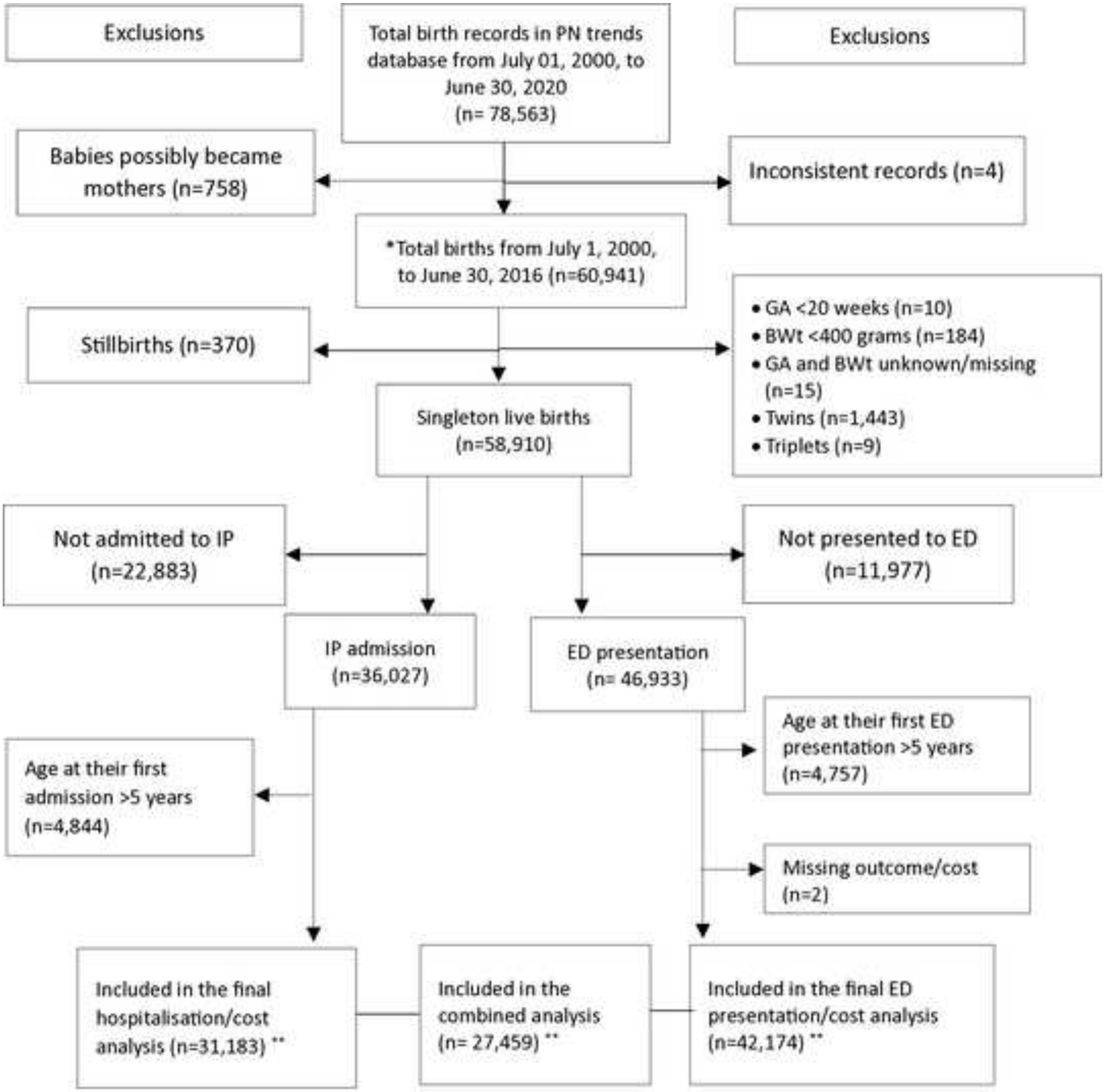

Fig 1. Cohort selection flow diagram, NT, Australia, 2000 to 2016.

Fig 2: Trends of adverse perinatal outcomes, NT, Australia, 2000 to 2016.

Click here to access/download;Figure;Fig 2. Trends of adverse perinatal outcomes.tif

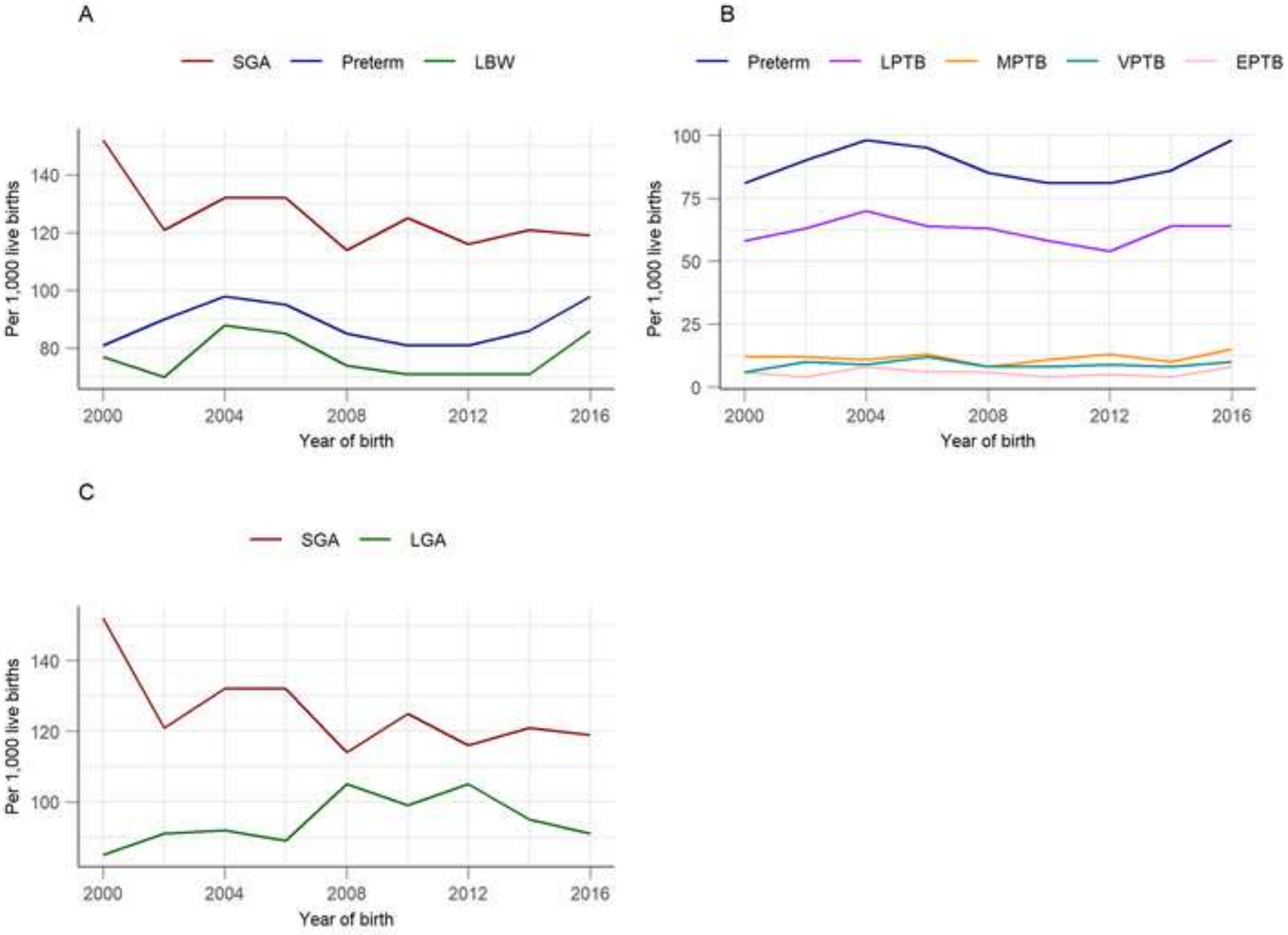

Fig 3. Trends of hospitalisation and ED presentation by adverse perinatal outcomes, NT, Australia, 2000 to 2020.

Click here to access/download;Figure;Fig 3. Trends of hospitalisation and ED presentation.tif

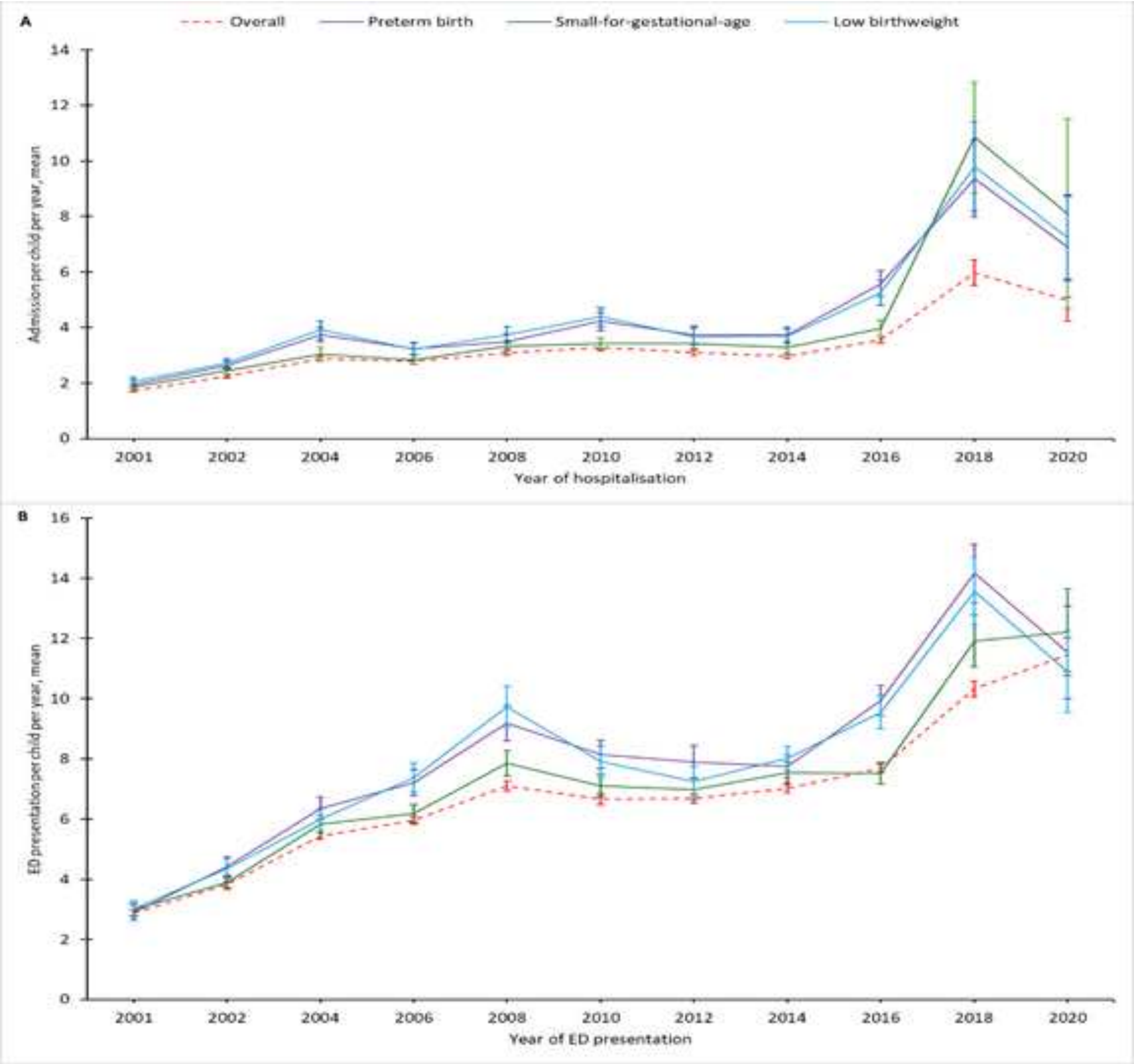

Fig 4. Hospitalisation cost by child age and adverse perinatal outcomes, NT, Australia, 2000 to 2020.

Click here to access/download;Figure;Fig 4. Hospitalisation cost by child age and outcomes.tif

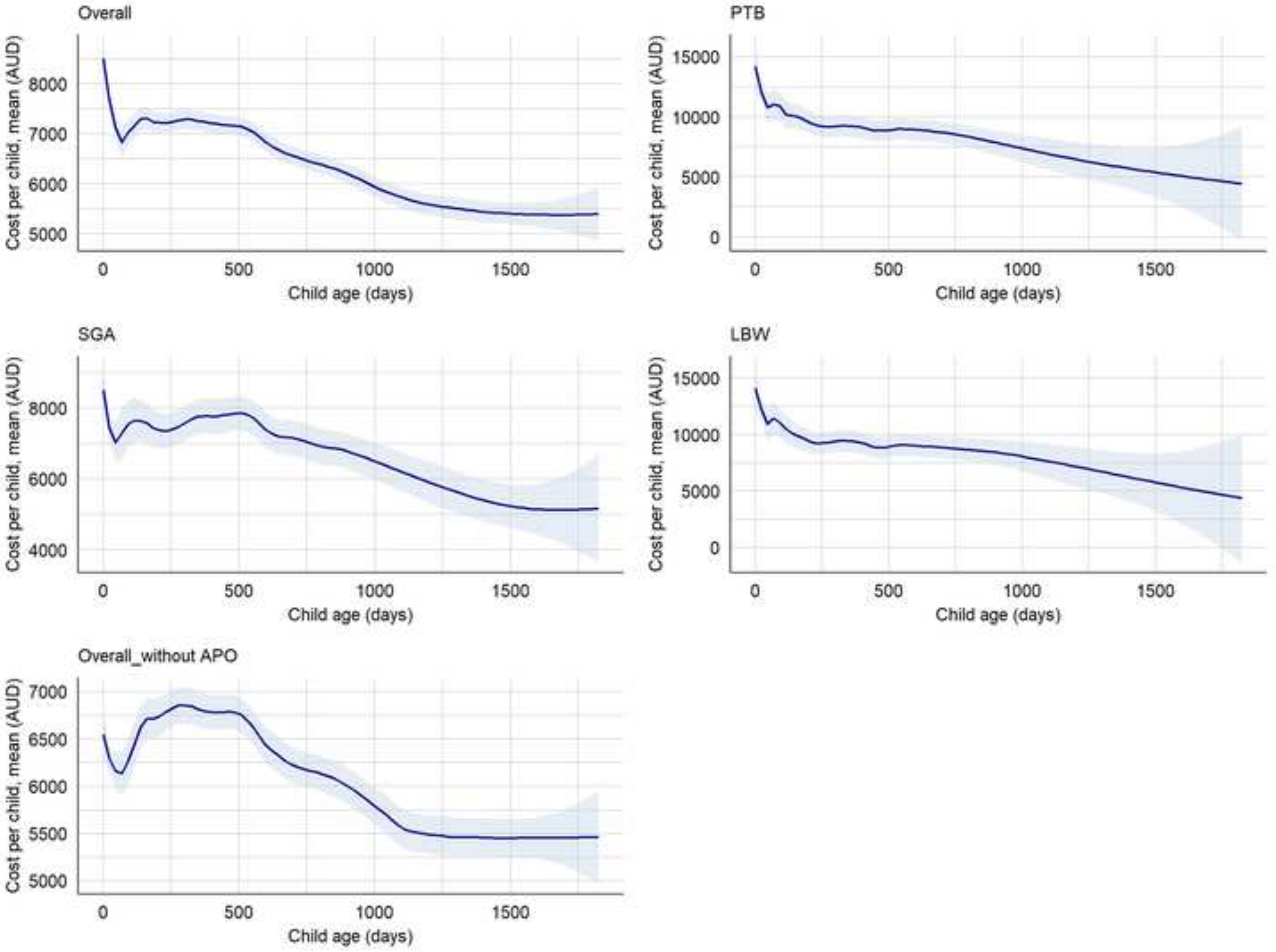

Fig 5. Trends of hospitalisation cost by adverse perinatal outcomes, NT, Australia, 2000 to 2020.

Click here to access/download;Figure;Fig 5. Trends of hospitalisation cost by adverse outcomes.tif

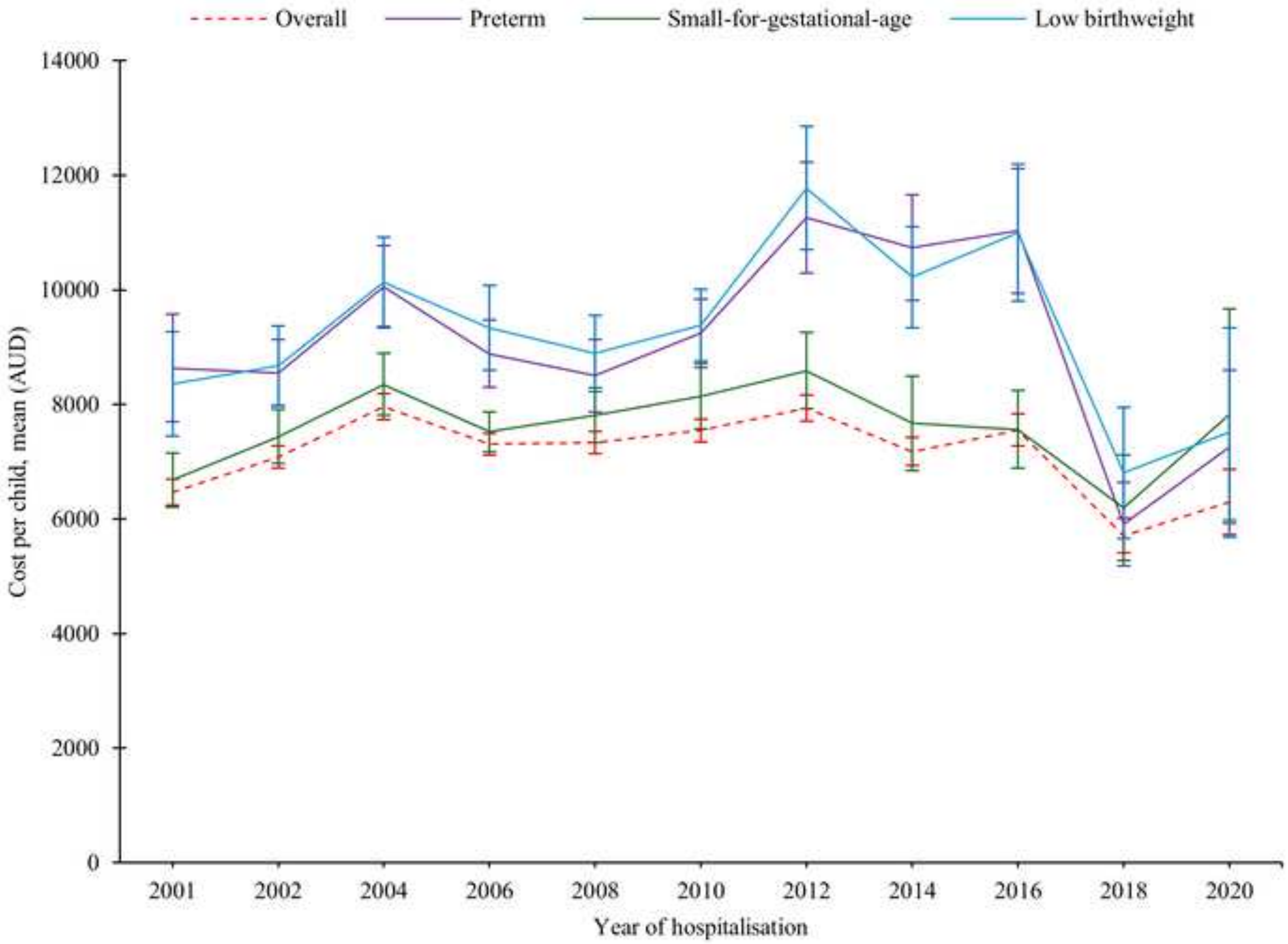

Fig 6. Cost of ED presentations by child age and adverse perinatal outcomes, NT, Australia, 2000 to 2020.

Click here to access/download;Figure;Fig 6. Cost of ED presentations by child age and outcomes.tif

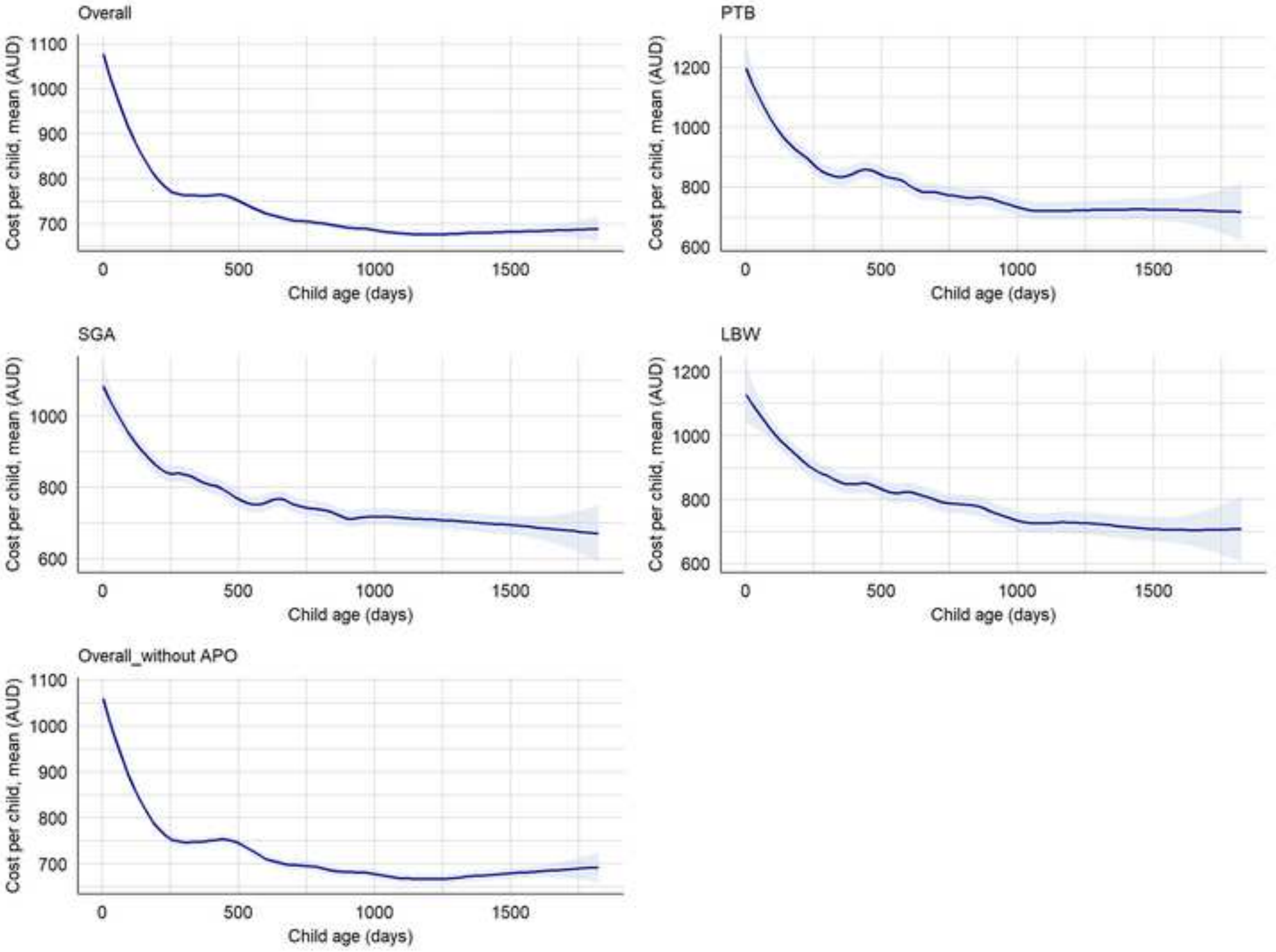

Fig 7. Cost of hospitalisation and ED presentation by child age and adverse perinatal outcomes, NT, Australia, 2000 to 2020.

Click here to access/download;Figure;Fig 7. Cost of hospitalisation and ED presentation.tif

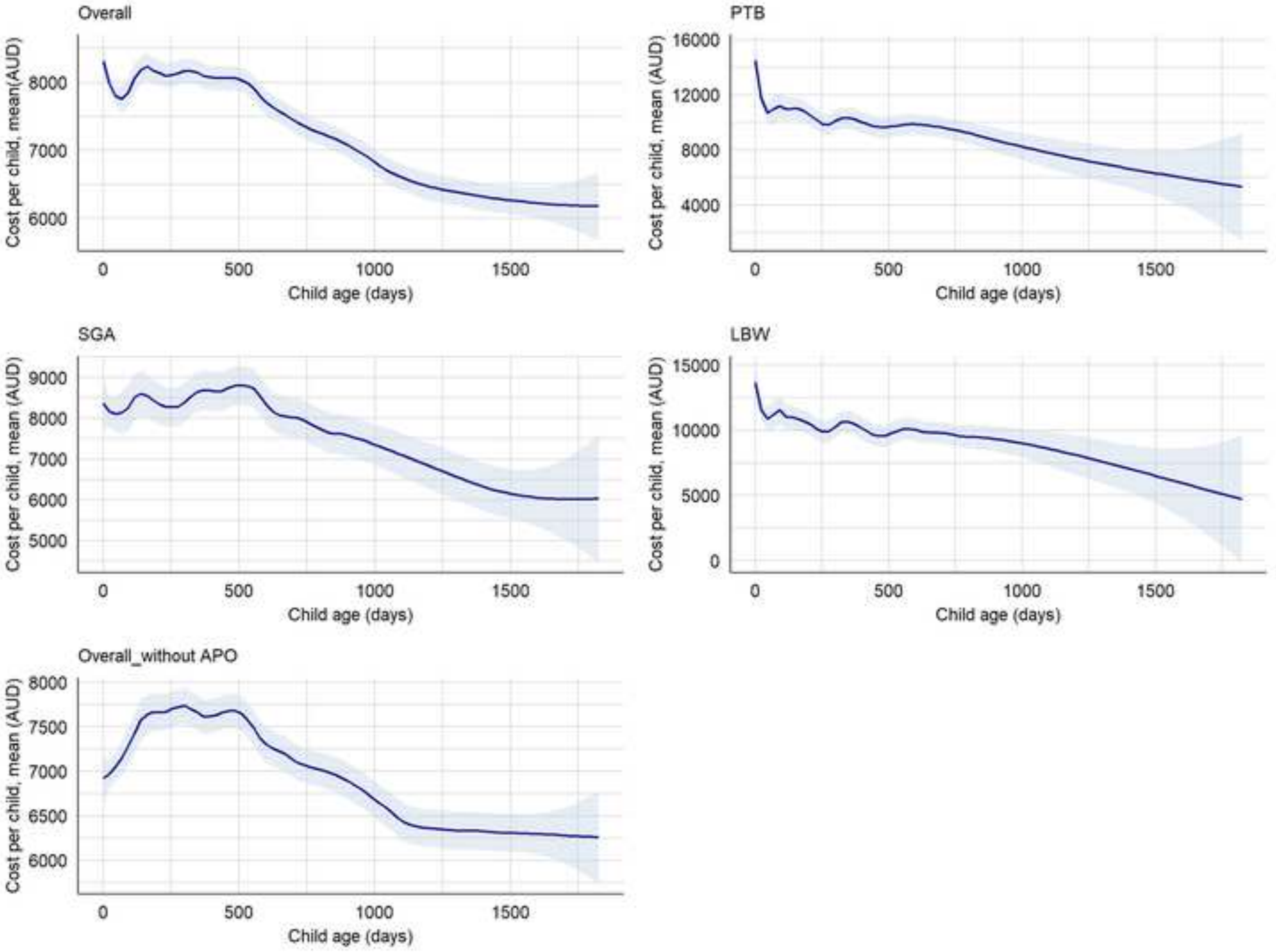

Fig 8. Non-linear relationship between hospitalisation cost and covariates, NT, Australia, 2000 to 2020.

Click here to access/download;Figure;Fig 8. Hospitalisation cost and covariates.tif

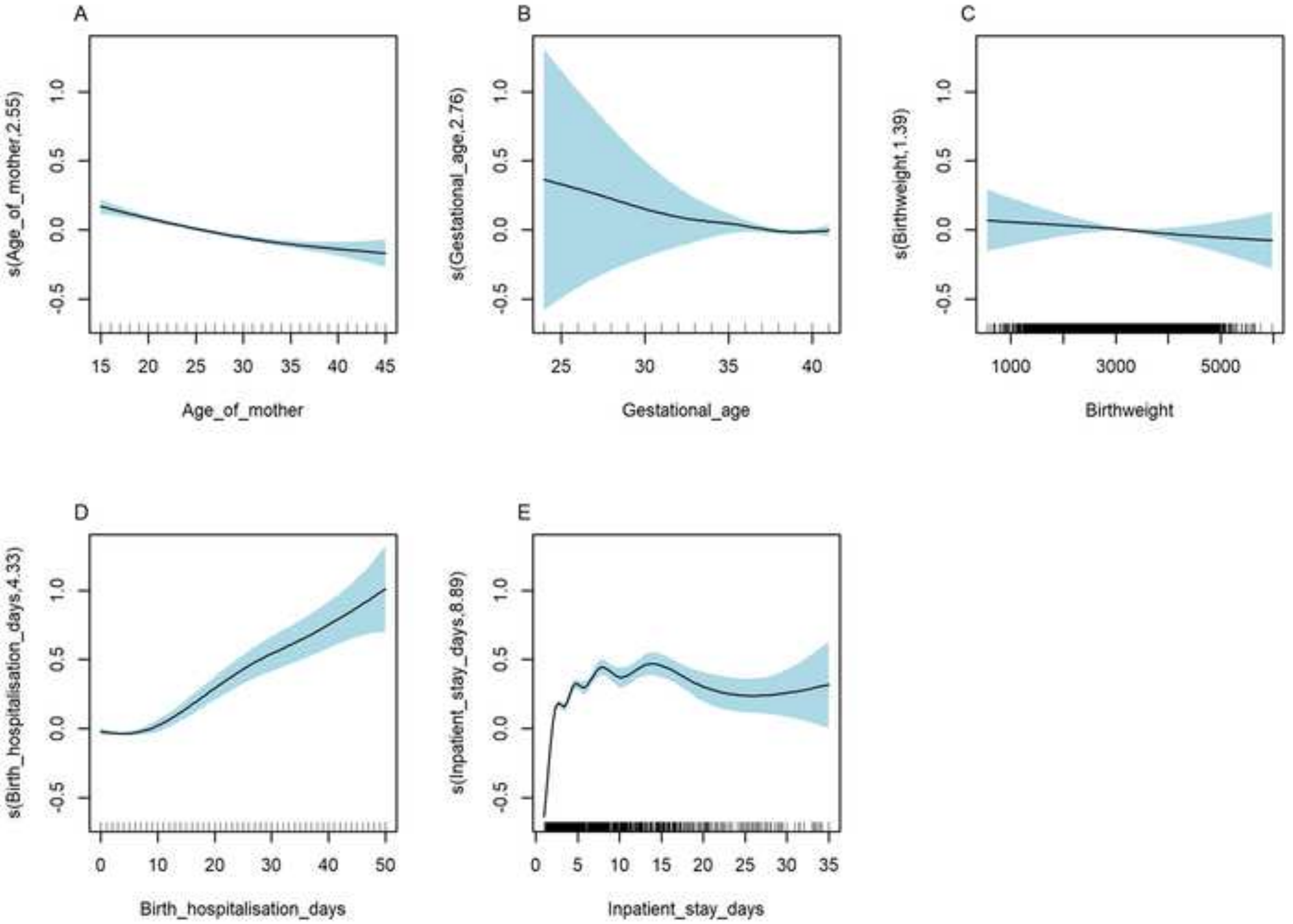

Fig 9: Non-linear relationship of cost of ED presentation and covariates, NT, Australia, 2000 to 2020.

Click here to access/download;Figure;Fig 9. Cost of ED presentation and covariates.tif

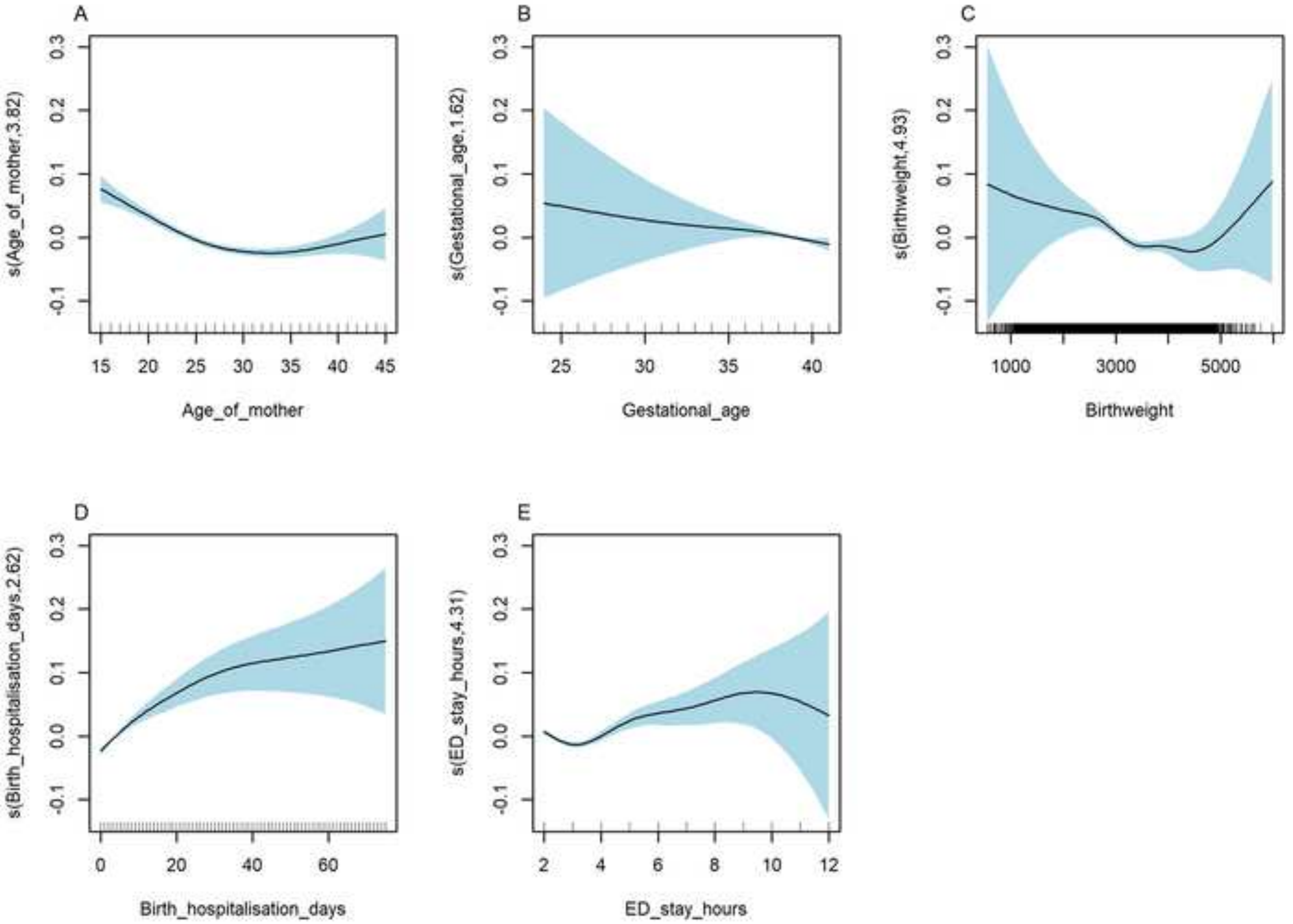

Click here to access/download
Supporting Information
Supplementary Figures_Haile et al..docx

Click here to access/download
Supporting Information
Supplementary Tables_ Haile et al..docx

Ethical approval letter (Original)

Click here to access/download
Supporting Information
HREC 22-4363_Approval Letter.pdf

Ethics approval letter (Reciprocal)

Click here to access/download
Supporting Information
Reciprocal Approved.pdf

Ethics approval letter (Amendment)

Click here to access/download

Supporting Information

HREC 22-4363 Amendment Approval Letter 08.08.23.pdf
