# Supplementary material for: Trends in adverse perinatal outcomes and associated hospitalisations, emergency department presentations, and healthcare costs from birth to early childhood in the Northern Territory, Australia: A two-decade population-based study
Source: PLOS Glob Public Health. 2025 Aug 7;5(8):e0004985. doi: 10.1371/journal.pgph.0004985 (PMC12331054; doi:10.1371/journal.pgph.0004985)
Supplement: Attachment — Submitted filename: Response to reviewers_Haile et al.,.docx [file pgph.0004985.s022.docx]

**July 1, 2025**

Shiyam Sunder, MBBS, MSc epidemiology, PhD

Academic Editor

*PLOS Global Public Health*

**Re: Response to reviewers – PGPH-D-25-00279**

Dear Editor,

We sincerely appreciate the opportunity to revise our manuscript, *Trends in adverse perinatal outcomes and associated hospitalisations, emergency department presentations, and healthcare costs from birth to early childhood in the Northern Territory, Australia: A two-decade population-based study*, for further consideration for publication in *PLOS Global Public Health*. We are grateful for the thoughtful and constructive comments provided by the reviewers, which have strengthened our work.

We have carefully addressed all comments and incorporated the suggested changes into the revised manuscript. A detailed point-by-point response outlining the revisions is provided in the following pages.

Thank you for your time and consideration. We hope the revised manuscript meets the journal’s standards and is acceptable for publication.

Kind regards,

**Authors – PGPH-D-25-00279**

**Manuscript Title:** Trends in adverse perinatal outcomes and associated hospitalisations, emergency department presentations, and healthcare costs from birth to early childhood in the Northern Territory, Australia: A two-decade population-based study

**Manuscript ID:** PGPH-D-25-00279

**Response to reviewers’ comments**

**Reviewer #1:** This manuscript addresses an important issue by examining the trends and healthcare burden of adverse perinatal outcomes. The authors should be commended for their use of a large, linked, population-based dataset and for employing advanced statistical methods to analyze cost drivers. Below are my specific few comments and suggestions.

**Authors:** Thank you for taking the time to review our manuscript and for your thoughtful and constructive comments. We have carefully considered all suggestions and have incorporated them in our revision; and provide further point-by-point response as follows.

Comment 1-The rationale for combining PTB, SGA, and LBW could be justified. These outcomes are interrelated but also distinct with different etiologies and implications.

**Response:** While PTB, SGA, and LBW are distinct perinatal outcomes, they are also interrelated. Our decision to analyse them both individually and as a combined outcome was based on several key considerations:

First, these outcomes frequently co-occur and share overlapping maternal and environmental risk factors, especially in high-risk populations. Second, from a health systems and policy perspective, PTB, SGA, and LBW collectively represent early-life vulnerabilities that are strongly associated with increased health service utilisation and cost burdens throughout early childhood. Third, creating a composite outcome allowed us to quantify the overall burden of adverse perinatal conditions, which is particularly relevant for informing service planning and resource allocation.

Importantly, we also conducted disaggregated analyses to reflect their individual impact and ensure that the nuanced differences in aetiology and outcome are not overlooked. We have clarified this rationale in the revised Introduction (page 4, lines 78–90), which now reads:

“Children born with adverse perinatal outcomes, such as PTB, SGA, and/or LBW, often require longer hospital stays, more frequent healthcare visits, and higher levels of intensive care. Consequently, they contribute to substantial healthcare resource utilisation and impose both short- and long-term economic burdens on the health system. PTB, SGA, and LBW are distinct clinical outcomes, each with unique aetiologies. However, these outcomes may co-occur and share common risk factors, including maternal health conditions, socio-demographic disadvantages, and environmental exposures. These conditions are also associated with similar long-term health consequences, such as increased morbidity, developmental vulnerabilities, and mortality (Eichenwald & Stark, 2008; Katz et al., 2013; van Hasselt et al., 2024). From a health systems perspective, PTB, SGA, and LBW represent a cluster of early-life adversities that place overlapping and sustained demands on healthcare services. To comprehensively assess the cumulative burden of adverse perinatal outcomes, a combined analytic approach that captures any instance of PTB, SGA, and/or LBW enables quantification of the total healthcare impact of these interrelated conditions. This approach is crucial for informing system-wide service planning and equitable resource allocation. Therefore, in this study we: (i) estimated the incidence and trends of adverse perinatal outcomes (PTB, SGA, and/or LBW); (ii) measured hospitalisation and emergency department (ED) presentation rates and trends; and (iii) analysed associated healthcare costs during the first five years of life over the past two decades, identifying the key drivers of these costs from a health systems perspective in the NT, Australia.”

Comment 2- The Eligibility criteria (Lines 129 to 134) lack clarity on congenital anomalies or neonatal death cases

**Response**: Thank you for the critical insight. We included all singleton live births from July 1, 2000, to June 30, 2021. We included congenital anomalies to show the full healthcare burden of adverse perinatal outcomes in the early childhood period. We have now included the revised statements in pages 5 to 6, lines 142 to 149:

“Our study cohort included all singleton live births from July 1, 2000, to June 30, 2016. This timeframe ensured a complete five-year follow-up by June 30, 2021, enabling comprehensive assessment of hospitalisation and ED costs during the critical early childhood. Births with a birthweight below 400 grams or a gestational age less than 20 weeks were excluded, as they were considered non-viable. Children with congenital anomalies and those who died following birth hospitalisation were included in the cohort to ensure full representation of the population and its associated healthcare burden.”

Comment 3-Model selection methods are briefly mentioned (Lines 157 to 158), but detail on model diagnostics is not provided.

**Response**: We have now elaborated on the model diagnostics used during model selection and specification. In addition to reporting model fit statistics (AIC, REML, and R-squared), we assessed residual patterns, smooth term confidence intervals, and concurvity to evaluate model assumptions and multicollinearity. We also conducted sensitivity analyses and cross-validation to assess model robustness and potential overfitting. These details have been incorporated into the revised Methods section on page 6, lines 167 to 176, and now reads:

“We used Generalised Additive Models (GAM) to identify the drivers of healthcare costs, allowing for non-linear associations with adverse perinatal outcomes (Hastie, 2017; Wood, 2017). Thin plate and cubic regression splines were applied to flexibly model smooth, non-linear relationships between costs and covariates. Model selection was based on comparison of the Akaike Information Criterion (AIC), residual maximum likelihood (REML), and R-squared values, with the gamma distribution and log link providing the best fit. Model diagnostics included examination of residual plots, smooth term uncertainty (via confidence intervals), and concurvity statistics to assess multicollinearity between smooth terms. We assessed overfitting through cross-validation and confirmed model stability through sensitivity analyses. Interaction effects were examined using tensor product smooths (ti() terms in the GAM framework) to capture non-linear interactions between key covariates, such as birthweight and gestational age.”

Comment 4-There is inconsistent reporting of missing data. Especially regarding values <6 (Line 245). How much data was affected?

**Response**: Thank you for your observation. We have revised the Table to improve the clarity of missing data reporting. Specifically, we have now included subtotals and calculated percentages where appropriate. In accordance with the Human Research Ethics Committee (HREC) guidelines, individual cell values less than six are suppressed to maintain confidentiality. The extent of missing data varies by variable, and the provided subtotals allow readers to infer the magnitude of missingness more clearly. Our revision is found in Table 1 on page 9.

Comment 5-Were interaction effects considered during analysis?

**Response**: Thank you for this important comment. Yes, interaction effects were considered in our analysis. Specifically, we evaluated interactions between key variables (e.g., gestational age and birthweight) using tensor product smooths within the Generalised Additive Model (GAM) framework. Interactions that improved model fit, assessed through Akaike Information Criterion (AIC), Restricted Maximum Likelihood (REML), and visual inspection of interaction surfaces, were retained in the final model. These methodological details have been added to the revised Methods section (page 6, lines 168 to 176) as follows:

“Thin plate and cubic regression splines were applied to flexibly model smooth, non-linear relationships between costs and covariates. Model selection was based on comparison of the Akaike Information Criterion (AIC), residual maximum likelihood (REML), and R-squared values, with the gamma distribution and log link providing the best fit. Model diagnostics included examinations of residual plots, smooth term uncertainty (via confidence intervals), and concurvity statistics to assess multicollinearity between smooth terms. We assessed overfitting through cross-validation and confirmed model stability through sensitivity analyses. Interaction effects were examined using tensor product smooths (ti() terms in the GAM framework) to capture non-linear interactions between key covariates, such as birthweight and gestational age.”

Comment 6-decreasing length of stay could lead to cost savings’ (Line 504) oversimplifies the issue. Shorter stays could increase readmissions or affect health outcomes also. This required rephrasing.

**Response:** Thank you. We have revised the statement to acknowledge the potential risks associated with shorter hospital stays, such as increased readmissions or adverse outcomes, and to emphasise the importance of safe discharge planning and continuity of care. The revised sentence (page 19, lines 532 to 536) now reads:

“Thus, enhancing perinatal health interventions to reduce the length of birth hospitalisation and subsequent admissions or ED presentations may contribute to healthcare resource savings. However, such efforts should be balanced with ensuring safety and continuity of care, as shorter hospital stays may increase the risk of readmissions or adverse health outcomes if not carefully managed (van Hasselt et al., 2024).”

Comment 7-Also consider including the limitation of potential misclassification bias due to missing exact birthdates (Line 485)

**Response:** Thank you for your valuable comment. We have now acknowledged the potential for misclassification bias resulting from the absence of exact birthdates in the dataset. In our analysis of the economic burden associated with perinatal outcomes among children aged 0 to 5 years, we estimated each child’s age at the time of hospital admission by subtracting their date of birth from the admission date. However, the dataset only included the month and year of birth, not the exact day. To standardise the age calculation, we assumed that all children were born on the first day of their reported birth month. While this approach allowed for consistent age calculation across cases, it would introduce a bias, particularly for newborns and infants, by artificially increasing their age.

For instance, if a child was actually born on June 30, but admitted to the hospital on July 1, in the same calendar year, our method would classify the child as one month old, despite their actual age being only one day. Consequently, this child would be incorrectly considered to have been alive for the full month prior to admission. This assumption could lead to the underestimation of the true economic burden, as any cost calculation for that "first month" would be inaccurately assumed to be zero, since the child was not yet born during that time.

In reality, had the child been correctly classified based on their actual age, they may have incurred non-zero hospitalisation costs. Thus, this limitation could lead to misclassification of age, underestimation of healthcare costs for true perinatal cases, and a potential distortion in the assessment of the economic burden of early-life health outcomes. This limitation has been addressed in the revised manuscript (page 19, lines 514 to –521), as follows:

"As the NT Perinatal Trends dataset share only the month and year of birth, child age was estimated using the first day of the recorded birth month. This approximation may have introduced misclassification bias in age-specific analyses. For example, infants born later in the month were assigned an earlier estimated birthdate, which could have artificially inflated their calculated age. As a result, hospitalisations occurring at the end of the neonatal period (e.g., at 28 days of life) for these infants may have been misclassified into older age categories due to this inflated age estimate. This misclassification may have led to an underestimation of healthcare costs."

Reviewer #2: Please see my comments below.

Comment 1. Page 1, abstract: it is stated "Mean hospitalizations increased for PTB (1.3 ± 0.7 to 6.9 ± 6.0), LBW (1.3 ± 0.6 to 7.2 ± 5.2), and SGA (1.2 ± 0.6 to 8.1 ± 15.1), while readmission durations decreased".

Are these measured in terms of days or weeks?

**Response**: Thank you for this important clarification request. We have revised the Abstract to specify that the "mean hospitalisations" refer to the average number/frequency of admissions per child from birth to five years of age; not a duration, while hospitalisation duration is measured in days and emergency department (ED) presentation duration is measured in hours. These details have been added to the Abstract on page 2, lines 27 to 31 for clarity and now reads:

“The mean number of hospitalisations per child from birth to age five years increased for those PTB (1.3 ± 0.7 to 6.9 ± 6.0), LBW (1.3 ± 0.6 to 7.2 ± 5.2), and SGA (1.2 ± 0.6 to 8.1 ± 15.1), while the average length of hospital stay decreased over time. Similarly, the mean number of ED presentations per child increased for PTB (1.3 ± 0.5 to 11.5 ± 10.7), SGA (2.2 ± 1.9 to 12.2 ± 11.5), and LBW (1.2 ± 0.2 to 10.9 ± 8.7) children.”

Comment 2. Page 7 author stated, "Examining the economic burden trends for children born with adverse perinatal outcomes throughout childhood and across populations can support efforts to enhance perinatal health interventions..."

What other populations were considered in the analysis?

**Response**: Thank you for your comment. In the analysis, we considered population with different sociodemographic backgrounds including Indigenous and non– Indigenous, and those living in remote and urban areas. This stratification allows us to gain a more comprehensive understanding of the economic cost of adverse perinatal outcomes and to inform more targeted and equitable perinatal health interventions. Our revision can be found on page 4, lines 91 to 97, and now reads:

“Understanding trends in the economic burden associated with adverse perinatal outcomes across early childhood, particularly among priority populations such as Indigenous and non–Indigenous children, and those residing in remote versus urban areas—can inform equitable resource allocation and guide the development of effective, cost-efficient perinatal health interventions. These findings also provide a foundation for estimating potential cost savings from initiatives aimed at reducing the incidence of adverse perinatal outcomes or improving perinatal health more broadly.”

Comment 3. Page 10. Please describe how missing data was handled in the study?

**Response**: We have now clarified our approach to handling missing data in the analysis. We conducted a complete case analysis by excluding observations with missing values for variables included in the regression models. The overall proportion of missing data was low; its exclusion was unlikely to introduce significant bias in our estimates. In accordance with Human Research Ethics Committee (HREC) requirements to maintain confidentiality, individual cell counts less than or equal to six were not reported. While this limits the ability to report exact frequencies for some variables, we have included subtotals and percentages in Table 1 to provide a clearer understanding of the extent of missingness. This information has now been added to the Methods section on page 8, lines 226 to 234, and reads as follows:

“We conducted a complete case analysis by excluding observations with missing values for the three main adverse perinatal outcomes, PTB, LBW, and SGA, as well as for variables included in the regression models. The frequency of missing data was low (<6 cases, representing <0.019%) and was unlikely to bias the results. To assess the robustness of our findings, we performed subgroup analyses based on the adverse perinatal outcomes: PTB, SGA, and LBW. Sensitivity analyses were also conducted to examine the impact of extreme values (outliers) for variables such as maternal age, gestational age, and the duration of hospitalisation and ED stays. For the ED time spent, we restricted the analysis to durations between 15 minutes and 24 hours, consistent with the National Emergency Access Target in Australia(Sullivan et al., 2016).”

Comment 4. Page 16, table #2. Others is one of the categories of marital status.

Can author describe the category "others"?

**Response**: We defined others for marital status category and described it in a footnote in Table 2. Accordingly, “others” included widowed, divorced, separated, or unknown.

Comment 5. Page 16, table #2. How parity is defined in the table.

**Response**: Thank you for your comment. We have now clarified the definition of parity in Table 2. Parity is defined as a numeric variable representing the number of previous births the mother has had, ranging from 0 (primiparous) to a maximum of 13 in our dataset. This clarification has been added as a footnote under Table 2.

Comment 6. Page 17, table#2. Mother's country of birth is categorized in Australia and others.

It would be interesting to see which countries; besides Australia, mothers were born in.

**Response**: Thank you for this insightful comment. In response, we have provided a breakdown of the maternal region of birth categories in the Table footnote, based on the distribution in our dataset. Others category included Southeast Asia/China, New Zealand/Oceania, Americas, Africa/Middle East, Rest of Asia, UK/Ireland, and Rest of Europe. This has been added to Table 2 (pages 13 to 14) for clarity.

**References**

Eichenwald, E. C., & Stark, A. R. (2008). Management and Outcomes of Very Low Birth Weight. *New England Journal of Medicine*, *358*(16), 1700-1711. <https://doi.org/doi:10.1056/NEJMra0707601>

Hastie, T. J. (2017). Generalized additive models. In *Statistical models in S* (pp. 249-307). Routledge.

Katz, J., Lee, A. C. C., Kozuki, N., Lawn, J. E., Cousens, S., Blencowe, H., Ezzati, M., Bhutta, Z. A., Marchant, T., Willey, B. A., Adair, L., Barros, F., Baqui, A. H., Christian, P., Fawzi, W., Gonzalez, R., Humphrey, J., Huybregts, L., Kolsteren, P., . . . Black, R. E. (2013). Mortality risk in preterm and small-for-gestational-age infants in low-income and middle-income countries: a pooled country analysis. *The Lancet*, *382*(9890), 417-425. <https://doi.org/10.1016/S0140-6736(13)60993-9>

Sullivan, C., Staib, A., Griffin, B., Bell Jr, A., & Scott, I. (2016). The four hour rule: the National Emergency Access Target in Australia, time to review, systematic literature review appendix. *Aust Health Rev*, *40*(3), 319.

van Hasselt, T. J., Wang, Y., Gale, C., Ojha, S., Battersby, C., Davis, P., Kanthimathinathan, H. K., Draper, E. S., & Seaton, S. E. (2024). Timing of Neonatal Discharge and Unplanned Readmission to PICUs Among Infants Born Preterm. *JAMA Network Open*, *7*(11), e2444909-e2444909.

Wood, S. N. (2017). *Generalized additive models: an introduction with R*. chapman and hall/CRC.
